# Calcium and Nitrogen Availability Controls Root Exudation in Hydroponically Cultured Barley

**DOI:** 10.1111/pce.70528

**Published:** 2026-04-14

**Authors:** Ibadete Denjali, Vijay Kumar, Joana Janzen, Marcus Persicke, László Cselényi, Refat Abdel‐Basset, Karl‐Josef Dietz

**Affiliations:** ^1^ Biochemistry and Physiology of Plants, Faculty of Biology Bielefeld University Bielefeld Germany; ^2^ Center of Biotechnology, CeBiTec Bielefeld University Bielefeld Germany; ^3^ W. von Borries‐Eckendorf GmbH & Co. KG Leopoldshöhe Germany; ^4^ Botany and Microbiology Department, Faculty of Science Assiut University Assiut Egypt

**Keywords:** amino acids, calcium, membrane stability, membrane transport, nitrate, root exudation

## Abstract

Root exudation is a key component of plant‐rhizosphere interactome. It is increasingly evident that root exudates influence rhizospheric microbial communities and in turn can benefit plants through improved resource allocation. However, how suboptimal nutrient availability relates to control of root exudation is poorly understood. This study explores effects of calcium and nitrogen availability on barley (*Hordeum vulgare* L.) root architecture and metabolism with focus on metabolite exudation. Upon depletion of NO_3_^‐^ from the medium (‐N), both sugar and amino acid exudation dropped rapidly within 6 h, but this fast effect was absent for amino acids if also Ca^2+^ was omitted from the medium (‐Ca/‐N). In contrast, Ca^2+^ depletion alone (‐Ca) led to a fourfold higher amino acid exudation. Further, a modulatory role for Ca^2+^ was evident, as the exudation varied in Ca^2+^‐channel inhibitor concentration‐ and treatment time‐dependent manner. Among the 51 detected exuded metabolites, N‐deprivation increased the release of specific sugars, i.e. sucrose, fructose, and cellobiose (1.6 to 8‐fold), with potential roles as chemoattractants. This pattern is reflected in the increased C/N ratio in ‐N‐exudates. The results show a predominance of nitrogen availability and a modulatory role of Ca^2+^ in tight regulation of root exudation in dependence of NO_3_^‐^ availability and metabolic state.

## Summary statement

- N and Ca^2+^ shortage affects barley root exudation in an opposite manner with strong effect on exudated metabolites, and validated by application of Ca^2+^ channel inhibitors.

## 1 Introduction

Global climate change threatens crop yields worldwide through diverse environmental stresses (Alotaibi 2023; Chowdhuri and Pal 2025). Fertilisation of soil, to improve crop yields, usually has negative consequences like soil acidification, calcium (Ca^2+^) displacement, as well as ground water contamination, contributing to environmental pollution and accelerating climate change (Dong et al. 2022b; McGrath et al. 2014; Tian and Niu 2015). A complex dynamics of multiple environmental factors including nutrient limitations drives plant productivity (Harpole et al. 2017) by synergistic, neutral or antagonistic interactions (Harpole et al. 2011; Lu et al. 2025; Olde Venterink 2016). Therefore, plant performance is determined by (i) feedback control of mineral nutrient uptake, (ii) relative mineral nutrient availability determined by nutrient crosstalk, as well as (iii) plant‐microbe interactions in the rhizosphere determined by the plant through dynamic adjustment of root exudation (Adeniji et al. 2025; Briat et al. 2020; Zhao et al. 2021).

Root exudation often is considered as a passive flow of metabolites along the concentration gradient between root cells and rhizosphere (Badri and Vivanco 2009; Canarini et al. 2019). The process depends on cell membrane permeability, cell integrity, compound size and polarity, nutritional status, and microbial interactions (Badri and Vivanco 2009; Canarini et al. 2019). The conditional release of metabolites can be used by plants as a signal to induce structural changes and for plant‐plant and plant‐microbe communication, raising questions for it being a passive or regulated process (Canarini et al. 2019; Wang et al. 2021).

Advancement in the mechanistic understanding of phloem unloading at the root tip (Ross‐Elliott et al. 2017), has provided a potential method for root exudation control, especially concerning primary metabolites. Undifferentiated root tip area, free of casparian strips and full of plasmodesmata, would allow for unrestricted symplastic and apoplastic flow of primary metabolites (sugars, amino acids (AA) and organic acids (OAs)) through cortex and epidermal cells and can be regulated via source‐sink activity (Canarini et al. 2019). Although small molecules like glycerol or urea can diffuse through membranes, large uncharged polar and all charged molecules require specific membrane transporters as facilitators (Baetz and Martinoia 2014; Yang and Hinner 2015), allowing transcriptional, translational and post‐translational control of exudation.

This transport involves the **U**sually **M**ultiple **A**mino Acids **M**ove **I**n and Out **T**ransporter (UMAMIT), **G**lutamine **Du**mper (GDU), **C**ationic **A**mino Acid **T**ransporter (CAT) for AAs (Besnard et al. 2016; Dinkeloo et al. 2018; Pratelli et al. 2009; Yang et al. 2010), **S**ugars **W**ill **E**ventually be **E**xported **T**ransporter (SWEET) family for sugars (Manck‐Götzenberger and Requena 2016), **AL**uminium‐activated **M**alate **T**ransporter (ALMT) and **M**ultidrug **A**nd **T**oxin **E**fflux (MATE/citrate) transporters for organic acids (Meyer et al. 2010). Besides these mostly passive transporters, H^+^‐coupled MATE‐type antiporters and **A**TP‐**B**inding **C**assette (ABC) transporters for secondary metabolites participate in active transport of root exudates (Kang et al. 2011; Vives‐Peris et al. 2020). Further, exocytosis through vesicles enables export of certain high molecular weight metabolites, e.g. in combating pathogen attack (Weston et al. 2012).

As a general rule, these transporters function in a substrate‐specific manner and export primary metabolites down their concentration gradient. The conditional nature of root exudation, the availability of a wide variety of transporters, and its apparent correlation to source‐sink relationships highlights the required regulation of this process and hence cannot just be a “passive” event (Canarini et al. 2019). Different activation speeds for anion channels, i.e. **SL**ow **A**nion **C**hannels (SLACs) and **QU**ick **A**nion **C**hannels (QUACs) are known (Dreyer et al. 2012), and could define the control depth. Regulation of exudation under both optimal and stress conditions coordinates resource distribution for shaping the rhizosphere, plant growth and defence.

The mechanistic understanding of this regulation is challenging and must consider multiple interacting factors. Root exudate quality and quantity is plant species‐specific and is modified by environmental conditions (Badri and Vivanco 2009; Chai and Schachtman 2022; Santangeli et al. 2024; Vives‐Peris et al. 2020). Several abiotic stresses like salinity, drought, heavy metals exposure and heat influence root exudate chemistry and recruit beneficial microbes aiding in stress acclimation (Ahlawat et al. 2024). Similar scenarios exist for nutritional deficit. For example, a conserved exudation of OAs like citrate, isocitrate, malate and succinate has been reported from 15 different eucalyptus species and also rape (*Brassica napus* L.) under P‐deficient conditions (De Andrade et al. 2022; Hoffland et al. 1992).

Under N‐deficiency, the release of a strigolactone, 5‐deoxystrigol, was shown for sorghum (*Sorghum bicolor* (L.) Moench), but not red clover (*Trifolium pratense* L.) (Yoneyama et al. 2012). N‐starvation in maize (*Zea mays* L.) however, leads to suppression of AA exudation (Carvalhais et al. 2011). Also, N‐limiting conditions, reflected in C/N ratio, increased flavonoid secretion that facilitated rhizobia symbiosis, enhancing N‐fixation (Liu and Murray 2016). In *Lupinus albus*, an N‐signalling and P‐starvation cross talk was revealed to control root exudation. Thus, tissue‐ and cell type‐specific NO‐accumulation positively correlates with citrate exudation under P‐limiting conditions (Wang et al. 2010). For micronutrient acquisition as well, increased exudation of phytosiderophores has been identified as a common strategy under both Fe‐ or Zn‐deficiency in wheat (*Triticum aestivum* L.) and barley (*Hordeum vulgare* L.) (Zhang et al. 1989).

A closer look at nutrient deprivation and root exudation studies shows a focus on N‐, and P‐starvation, but not on metallic nutrients like Ca^2+^, K^+^ and Mg^2+^ (Sardans et al. 2023). A few studies carried out in rice (*Oryza sativa* L.), or tree species like *Picea glauca*, *Abies lasiosacarpa* and *Cryptomeria japonica* highlight significant modulation of root exudation under lowered K^+^, Ca^2+^ and Mg^2+^ (Liu et al. 2011; Ohta and Hiura 2016; Tuason and Arocena 2009). Ca^2+^, as a secondary messenger, has been established as critical component of abiotic and biotic stress signalling pathways, and is crucially involved in the regulation of diverse nutrient deficiencies through management of plant ion homoeostasis (Wang et al. 2023; Wilkins et al. 2016). Cytosolic Ca^2+^ transients indicate stress‐specificity through variation in duration, amplitude, frequency, and spatial distribution through rapid and regulated Ca^2+^ influx through channels and efflux via Ca^2+^ transporters (Kudla et al. 2018; Tian et al. 2020; Wang et al. 2023).

The versatility of Ca^2+^ as a signal is possible due to a wide variety of transport mechanisms along with a high specificity and affinity of a myriad of Ca^2+^‐binding proteins (Luan and Wang 2021). This dependence on Ca^2+^‐signalling is not restricted to NO_3_⁻ only, but extends of most nutrients (Wang et al. 2023). The list of ion fluxes regulated by **C**alcineurin **B**‐**L**ike proteins (CBL)‐**C**BL **I**nteracting **P**rotein **K**inase (CIPK) is still increasing (Dong et al. 2022a). As structural component of the plant cell wall and plasma membrane, Ca^2+^ contributes to membrane stability and permeability (Jing et al. 2024; Thor 2019). Ca^2+^ fluxes across the membrane alters membrane potential (Wdowiak et al. 2024), in turn affecting H^+^‐ATPase pumps and thus proton gradients and ion uptake (Dong et al. 2022a; Nietfeld and Prenzel 2015). Therefore, the calcium influence on membrane permeability is multifaceted: it fortifies the membrane structure, orchestrates signalling that governs transporter activity, and maintains the delicate balance required for optimal ion exchange and potentially metabolite exudation in plant roots.

This study explores the integrated response of barley to Ca²⁺ and NO₃⁻ deficiency, recognising shared and interdependent signalling pathways. Our research focused on how these deficiencies influence nitrogen assimilation, root exudation, and potentially root plasticity. The results demonstrate that barley roots respond differently to individual and combined nutrient stress, with notable adjustments in root exudate composition. Nitrogen starvation appears to be a dominant factor in the control of root exudation, masking Ca^2+^‐starvation effects on membrane permeability and transport. The role of Ca^2+^ as a nutrient and signal is pharmacologically tested. These findings offer novel insights into the mechanisms of plant acclimatisation through adjustment of root exudation profile under differential nutrient limitations.

## 2 Material and Methods

### 2.1 Plant Growth and Root Exudation

*Hordeum vulgare* L. cv. SU MIDNIGHT (Winter barley; W. von Borries‐Eckendorf GmbH & Co. KG, Leopoldshöhe, Germany) seeds were used in this study. Seeds were mixed with vermiculite and soaked with lukewarm water for 30–40 min. Excess water was decanted off and seeds were first germinated in covered pots at 21°C at 10 h/19°C for 14 h. After 3 days of dark incubation germinated seedlings with 4–6 embryonic roots were selected and transferred to 1.7 L hydroculture pots containing modified Hoagland's nutrient solution (Macronutrients: 1.5 mM KCl, 0.5 mM MgCl_2_, 1.0 mM CaCl_2_, 3.5 mM NaNO_3_, 0.25 mM Na‐Pi (pH 5.5), 0.5 mM Na_2_SO_4_ and micronutrients: 12 µM Fe(III)‐tartrate, 11.5 µM HBO_2_, 0.075 µM CuCl_2_, 0.2 µM ZnCl_2_, 1.25 µM MnCl_2_, 0.025 µM Na_2_MoO_4_) and were fixed on mashed net with two O‐rings. Seedlings were grown for 5 days in a short‐day growth chamber (21°C/19°C light/dark, 55% RH, and light intensity of 100 μmol photons m⁻² s⁻¹). At seedling age of 8 days, healthy seedlings were transferred to fresh nutrient media and subjected to a 10 days treatment regime: Control (normal media), ‐Ca (Ca‐deficiency), ‐N (N‐deficiency), and ‐Ca/‐N (combined Ca and N deficit). Plant growth phenotype was recorded and fresh weight measured after 10 d treatment. Root growth imaging was carried out on ChemiDoc MP Imaging System (Bio‐Rad, Germany). At different treatment times, fresh plant material was harvested, rapidly frozen using liquid N_2_ and stored at ‐80°C until further use.

For exudate quantification after the different treatment time points, seedlings were removed from the nets, roots washed with distilled water, gently dried, and transferred to 25 mL conical tubes (five seedlings in 25 mL media). Seedlings from each treatment were transferred to the same media condition in the conical tubes. A 6 h incubation under the same growth chamber conditions allowed root exudate collection in 25 ml volume from intact seedlings. It was ensured that only the roots were submerged in the nutrient solution, while the seeds and shoots remained above, fixed to one corner with an aluminium foil cover. The 6 h time period allowed sufficient metabolites quantities for further analysis, and was short enough to avoid cell contents from sloughed‐off root cells (Carvalhais et al. 2011). In order to rule out inadvertent effects of missing Na^+^ in minus NaNO_3_ and missing Cl^‐^ in minus CaCl_2_ conditions, root exudation was also measured after supplementation with 3.5 mM NaCl for ‐N, 2 mM NaCl for ‐Ca or 5.5 mM NaCl for ‐Ca/‐N conditions.

Root fresh weight was recorded and collected root exudates were filtered using sterile syringe filters (*Rotilabo, PVDF*, with a pore dimension 0.22 µM) to remove any particles. Further, either fresh or freeze‐dried root exudates were quantified for different metabolites, electrical conductivity, K^+^ and UV spectra. To investigate the role of Ca^2+^ in modulating sugar and AA exudation, roots were exposed to calcium channel blockers, i.e. verapamil (0.1 or 0.5 µM) and lanthanum (LaCl_3_; 0.1 or 0.5 µM) for 6 h (d 0) or full treatment duration (d 10). The applied inhibitor concentrations were selected based on preliminary experiments scrutinising the inhibitor effects on growth using a wide concentration range (Figure S10). Cell sap osmolarity was quantified in shoot and root using semi‐micro osmometer A0800 (Knauer, Germany).

### 2.2 Nitrate Reductase Activity

Nitrate reductase (NR) activity was measured for all treatment conditions after 3 and 10 d of treatment using a spectrophotometric assay (Kim and Seo 2018). For enzyme extraction, 100 mg of powdered plant tissue was homogenised in 150 µL of extraction buffer (250 mM Tris‐HCl (pH 8.0), 1 mM EDTA, 1 µM Na₂MoO₄, 5 µM flavin adenine dinucleotide (FAD), 3 mM dithiothreitol (DTT), 12 mM 2‐mercaptoethanol, 1%(w/v) bovine serum albumin, and 250 µM phenylmethylsulfonylfluoride; PMSF) in a 2 mL screw‐cap tube containing 1 mm zirconia beads in a Precellys homogeniser (1 × 5800 rpm; 2 × 15 s, 30 s pause, and 1 × 6800 rpm; 3 × 20 s, 30 s pause, at 4°C–8°C). After homogenisation, the samples were centrifuged in two sequential steps at 16,000 x *g*, 4°C, for 10 min each to collect particle‐free supernatant. For NR activity, 10 µL of protein extract was mixed with 3.4 µL of 10 mM NADH, and 166.6 µL of reaction buffer (100 mM Na‐Pi, pH 7.5, added with 40 mM NaNO_3_). The assay mixture was incubated at 25°C for 5 min. To terminate the reaction, 40 µL of 1% sulphanilamide (in 3 M HCl) and 40 µL of 0.05% N‐(1‐naphthyl)ethylenediamine hydrochloride were added. After a further 15 min incubation in the dark at 25°C, absorbance was measured at 540 nm. Nitrite standards (0‐400 µM) were used in parallel to quantify nitrite amount generated in the reaction. Absorbance was read at 540 nm and specific NR activity was expressed as nitrite formed in nmol h⁻¹ mg⁻¹ protein. Protein amount was quantified separately using the Bradford assay.

### 2.3 Ion Leakage: Analysis of K^+^‐Contents and Conductivity

Conductivity of the collected barley root exudates was measured using conductivity metre (ECTestr low^+^, Eutech Instruments, Singapore). For sample measurements, 1 mL of fresh root exudate was applied to the conductivity electrode, and the values recorded were expressed in microSiemens (µS cm^−1^). Conductivity values were corrected based on the differences in root fresh weights under different treatments.

K^+^ content in fresh root exudates after 6 h incubation was determined using a flame photometer (Model 410; Sherwood Scientific Ltd., UK). Calibration was performed using K^+^ standard (1000 ppm K, Sherwood Scientific Ltd., UK) prepared in the range of 0–10 ppm. For the analysis, root exudate samples were diluted 1:10 with ultrapure water. To account for background K^+^ levels, the concentration measured prior to incubation was subtracted from the concentration after 6 h of incubation. The resulting net K^+^ release was then normalised to the fresh weight (FW) of the seedling roots used for exudate collection. Final exudated K^+^ amounts were expressed in µmol/g FW.

Ionic and metabolic signature of collected exudates under different treatments was also analysed through UV spectra in the range of 190 to 280 nm using a spectrophotometer (UV‐2401PC, UV‐VIS spectrophotometer, Shimadzu, Germany). For each sample, 1 mL of fresh root exudate was used for spectral analysis. Recorded spectra were corrected for the background signal of the respective media and normalised to the fresh weight of the roots used for exudation.

### 2.4 Sugar Quantification

Soluble sugar content in fresh plant tissue and root exudates was determined using a spectrophotometric anthrone assay (Yemm and Willis 1954). Extraction from the plant tissue (± 25 mg) was carried out in 1 mL 80% EtOH. Samples were incubated at 50°C for 10 min before homogenisation using Precellys homogeniser (5800 rpm, 2 × 15 s, 30 s pause) using silica beads. Homogenised samples were centrifuged for 5 min at 16,000 × g (4°C) and clean supernatant was collected. The assay required 100 µL of freshly collected root exudate or 30 µL of tissue extract which was mixed with 900 or 970 µL of freshly prepared anthrone reagent, respectively, in 1.5 mL Safe‐Lock microcentrifuge tubes. The samples were incubated in a water bath at 95°C for 15 min, after thorough mixing. Following incubation, the reaction was terminated by placing the tubes on ice for 5 min. A 200 µL aliquot from each reaction mixture was transferred to a 96‐well microplate, and the absorbance was measured at 620 nm using a microplate reader. The sugar content was calculated using a d‐glucose (0.5–50 µg) standard curve and normalised to root fresh weight (µmol g^‐1^ FW).

### 2.5 Amino Acid Quantification

AA contents in plant tissue and fresh root exudates were determined using a spectrophotometric Ninhydrin‐based assay (Stauß et al. 2024). AA extraction from about 50 mg frozen and powdered plant tissue was carried out in 1 ml 80% EtOH. Samples were homogenised in a 2 mL screw‐cap tube containing 1 mm zirconia beads in Precellys homogeniser as described above. Clean supernatant was collected using 2x centrifugation for 10 min each at 16,000 x *g* (4°C). Further, 400 µL freshly collected root exudate/plant extract was mixed with 600 µL of Ninhydrin reagent and mixture incubated at 90^°^C for 45 min. Following incubation, the reaction was stopped by placing the tubes on ice for 5 min. Samples were diluted 1:6 with 2‐propanol:water (1:1 v/v). A 200 µL aliquot from each reaction mixture was transferred to a 96‐well microplate, and the absorbance was measured at 570 nm using a microplate reader. The AA content was calculated using a l‐alanine (0.008–0.4 µmol) standard curve and normalised to root fresh weight (µmol g^‐1^ FW).

### 2.6 Metabolite Profiling

Metabolite quantification in leaf or root tissue, and in collected root exudates was performed using gas chromatography–mass spectrometry (GC‐MS) (García‐Caparrós et al. 2022; Wegener et al. 2023). Flash frozen plant tissue was powdered under liquid nitrogen. The powdered plant material along with frozen root exudates was freeze‐dried at −50°C. For metabolite extraction, about 5 mg freeze‐dried material was weighed into 2 mL screw‐cap tubes prefilled with 0.5 g of 0.5 mm zirconia beads. Samples were homogenised in 1 mL of 80% MeOH containing 10 μM ribitol as an internal standard, using Precellys homogeniser as above. The homogenates were then centrifuged at 21,000 × *g* for 20 min at room temperature, and 600 µL of the supernatant evaporated under a stream of nitrogen. Details pertaining to derivatization, GC‐MS analysis, and compound identification are available in cited literature (García‐Caparrós et al. 2022; Wegener et al. 2023). Metabolite abundance was expressed in relative units (r.U.) per gram of plant dry weight. For exudate samples, the root weight of the five seedlings under respective conditions was used for normalisation. *MetaboAnalyst* (v5.0 and 6.0) was used for the principal component analysis of the tissue and exudate metabolites (Pang et al. 2024; Pang et al. 2022; Xia et al. 2009; Xia and Wishart 2011). Further presentation of metabolites is described in respective figure legends.

### 2.7 C and N Analysis

Freeze dried plant tissue, and root exudates were further analysed for C‐ and N‐contents. Dried material (±4 mg) was transferred to tin capsules and subsequently analyzed using an element analyzer (Unicube, Elementar, Langenselbold, Germany). Sulphanilamide (C_6_H_8_N_2_O_2_S) was used as the reference compound.

### 2.8 K^+^ and Ca^2+^ Analysis in Root and Shoot

K^+^ and Ca^2+^ contents in barley roots and shoots were determined using a flame photometer (Model 410; Sherwood Scientific Ltd., UK). For sample preparation, 100 mg of dried plant tissue were weighed in 2 mL screw‐cap Eppendorf tubes. One millilitre of concentrated nitric acid (HNO₃) was added to each tube, and samples were homogenised using Precellys homogeniser as described above. Samples were incubated at 50°C for 2 h and, thereafter, centrifuged at 16,000 *x*g at 4°C for 10 min. The supernatant was collected and diluted with deionized water as required. The flame photometer was calibrated using 1000 ppm K^+^ and Ca^2+^ stock solutions (Sherwood Scientific Ltd., UK). To maintain measurement accuracy, flame photometer tubing was flushed with deionized water every 5 samples along with precision test using 10 ppm standard every 10 samples.

### 2.9 Statistical Analysis

Data are mostly presented (if not specified otherwise) as the mean ± standard error (SE) from multiple independent growth regimes and experiments per treatment (specified in figure legends). Statistical significance of the measured variables was evaluated using one‐way analysis of variance (ANOVA) to compare the means across the four tested treatments, followed by Tukey's post hoc test if required (IBM SPSS Statistics 20.0). Student's *t*‐test was only used for cases with two means. All analyses involved 10 seedlings per pot during the treatment period. Root exudation data was compared using Welch‐ANOVA and Games‐Howell post hoc analysis.

## 3 Results

N‐ and Ca^2+^‐deprivation and their combination (‐Ca/‐N) were applied to barley seedlings and scrutinised for effects on root growth and exudation rates of soluble sugars, AAs as well as a deep GC‐MS analysis for identifying a broad spectrum of exuded metabolites. Further, a pharmacological analysis involving Ca^2+^‐channel inhibitors was carried out to identify Ca^2+^‐dependence of root exudation under the evaluated treatment conditions.

### 3.1 Nutrient Deprivation and Plant Growth

**Figure 1:**
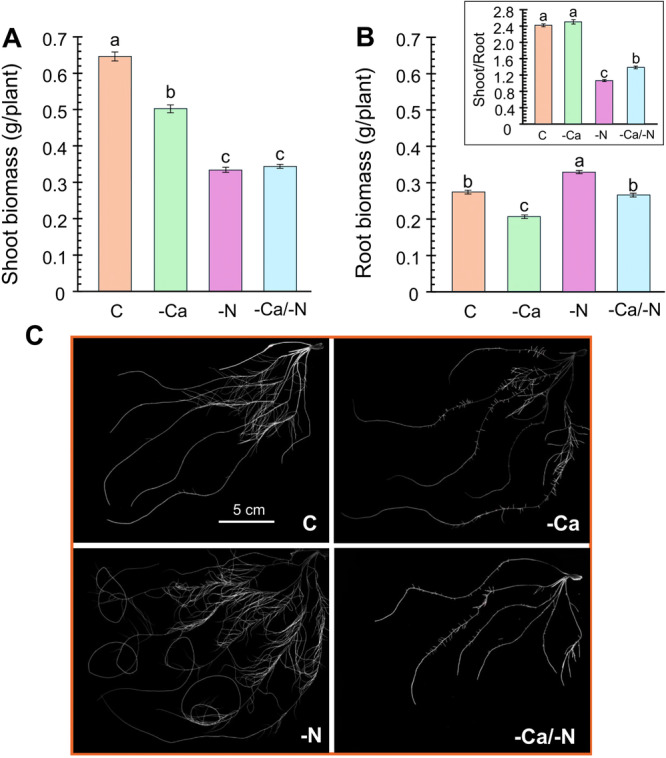
Barley seedling biomass in ‐N, ‐Ca and ‐Ca/‐N conditions. Barley seedlings (*Hordeum vulgare* L. cv. SU MIDNIGHT; winter barley) were grown in hydroponics at ‐N, ‐Ca and ‐Ca/‐N for 10 days. Seedlings were germinated on vermiculite and grown on normal growth media before transfer to treatment conditions at 8 days age. After 10 days of growth, shoot (A) and root (B) fresh weights were recorded (shoot/root ratio in the box) and root growth phenotype documented (C). Values presented are means ± SE (*n* = 12, 100–130 plants in total per treatment, one way ANOVA, Tukey's post hoc test, *p* < 0.05 for significant differences). Different letters denote statistical significance of differences among the four treatments. Control samples grown on full strength media are labelled as “C”. Further, growth analysis of barley seedlings on a wider array of N (N1: 3.5, N2: 0.58, N3: 0.016, and N4: 0 mM), and Ca^2+^ (Ca1: 1, Ca2: 0.0625, Ca3: 0.005, and Ca4: 0 mM) concentration combinations are presented in Figures S1, S2.

The deficiency‐specific changes in plant growth expectedly revealed stronger inhibition upon N‐ than Ca^2+^‐deprivation. Thus, shoot fresh weight decreased by about 50% in ‐N and 23% in ‐Ca condition (Figure 1A). The typical N‐starvation‐dependent stimulation of root branching, seminal root length and, therefore, increased investment of resources in root biomass or towards increased root surface was also evident from the 1.6‐fold higher root fresh weight for ‐N plants (Figure 1B,C) (Sun et al. 2020). The stimulation was lost in the combined ‐Ca/‐N‐starved plants, where average root biomass decreased by 20% (not significantly different from control) (Figure 1B,C). The same is reflected in shoot/root ratio as well. For wider understanding of this Ca/N‐interaction, tested multistep combinations of Ca, and N‐dilutions revealed that only complete absence of Ca led to this significant reversal of N‐starvation‐induced root branching and biomass accumulation, at most N‐dilution levels (Figures S1 and S2). For example, even at 216‐fold diluted Ca in nutrition media, especially in complete absence of N, increased root branching was apparent (Figures S1 and S2).

### 3.2 Nitrogen Assimilation, and C‐ and N‐Contents

**Figure 2:**
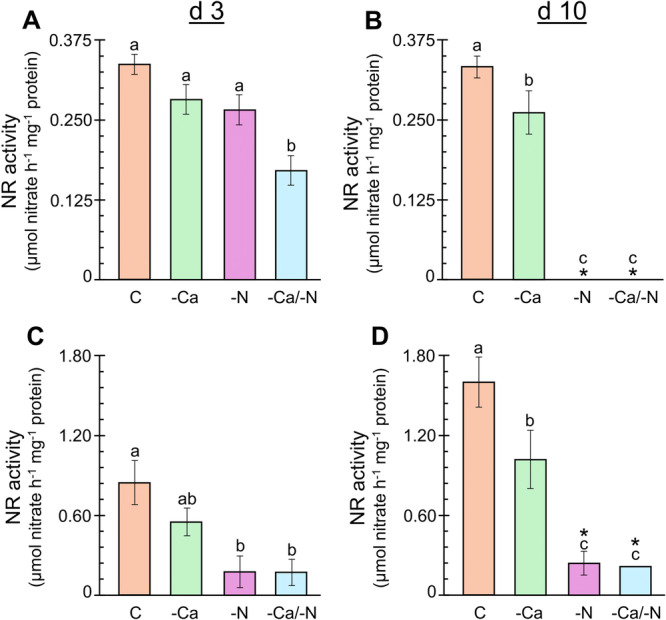
Effects of ‐N, ‐Ca and ‐Ca/‐N on nitrate reductase activity over the treatment period. Barley seedlings were grown in hydroponics with ‐N, ‐Ca and ‐Ca/‐N for 10 days. Seedlings were germinated on vermiculite and grown on normal growth media before transfer to treatment conditions at 8 days age. On Day 3 (A, C), and Day 10 (B, D) of treatment plant shoots (A, B) and roots (C, D) were harvested and analysed for nitrate reductase (NR) activity. Values presented are means ± SE (*n* = 9, three independent experiments, one way ANOVA, Tukey's post hoc test, *p* < 0.05 for significant differences). Different letters denote statistical significance of differences among the four treatments. Control samples grown on full strength media are labelled as “C”. Asterisks mark NR activity values which were too low to be detected and/or only a few measurements were possible. Further analysis of C‐ and N‐contents and changes in C/N ratio in a wider array of N (N1: 3.5, N2: 0.58, N3: 0.016, and N4: 0 mM), and Ca^2+^ (Ca1: 1, Ca2: 0.0625, Ca3: 0.005, and Ca4: 0 mM) concentration combinations on barley are presented in Figure S4. [Color figure can be viewed at wileyonlinelibrary.com]

As the first enzyme in nitrate assimilation, nitrate reductase (NR) activity was evaluated in shoots and roots of treated plants. Although no significant changes in total tissue N or soluble protein content were apparent after changing Ca‐availability (Figures S3, S4), NR activity was reduced in ‐Ca by 18%–22% (shoot) and 35%–37% (root) after Day 3 and Day 10, respectively (Figure 2A–D). The recorded changes in shoot and root after 10 d of treatment were statistically significant (Figure 2B,D). NR activity increased by ~1.9‐fold in roots from Day 3 to Day 10 for both control and ‐Ca‐conditions (Figure 2C,D), while under N‐deprivation, shoot NR activity was maintained at 79% of control after d 3, but dropped below detection limits towards d 10. Root NR activity was significantly reduced (80%–85%) in roots after Day 3 and Day 10 due to N‐starvation (Figure 2C,D). For the combined deprivation (‐Ca/‐N), NR activity followed the pattern of ‐N treatment, except at Day 3 in shoot. Interestingly, while NR activity decreased by ~ 20% for both ‐Ca and ‐N in shoot after Day 3 (not statistically significant), a significant 49% reduction was apparent for the combined starvation (Figure 2A). Shoot nitrate dropped gradually over 10 days of treatment for control and ‐Ca, while reduction was faster for ‐N and ‐Ca/‐N, reaching below detection limits by Day 6 (Figure S3E).

Similar to nitrogen contents, carbon contents did not vary significantly with changed Ca^2+^ supply. However, with increasing Ca^2+^‐depletion, a significant increase in root carbon content was evident, especially at optimal N‐supply and in 1:6‐dilution (Figure S4). Further, in roots at optimal N‐availability, a minor reduction in N upon Ca‐depletion was also reflected in changed C/N ratio (Figure S4). Only minor changes in shoot Ca^2+^, and K^+^ content were evident (Figure S4). A trend towards decreasing internal Ca^2+^‐levels with step wise reduction in external Ca^2+^‐availability developed along with a general drop in both Ca^2+^ and K^+^ contents at all higher N‐dilutions in root (Figure S5).

### 3.3 Root Exudation: Interaction of N‐ and Ca‐Deprivation

**Figure 3:**
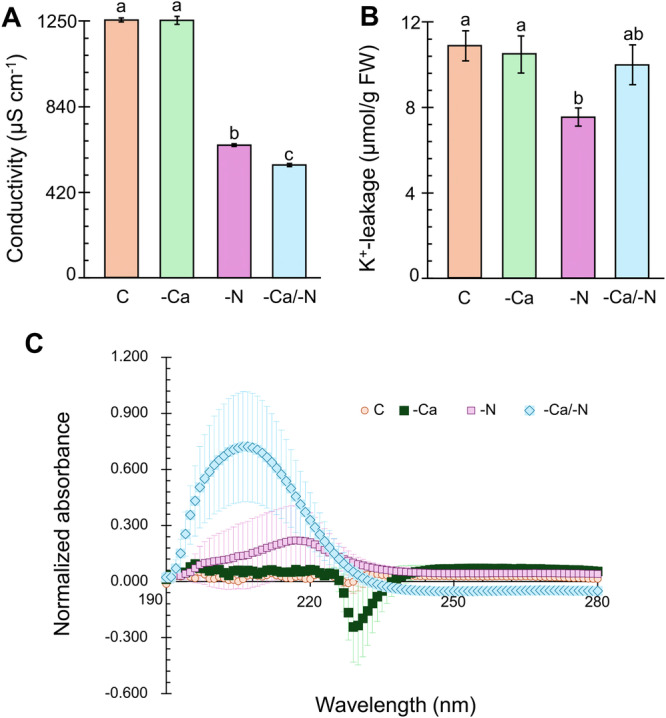
Primary analysis of barley root exudates under ‐N, ‐Ca and ‐Ca/‐N. Hydroponically grown barley after 10 days of exposure to ‐N, ‐Ca and ‐Ca/‐N were analysed for root exudation. Root exudates were collected in freshly prepared nutrient media, of respective nutrient availability, on d 10 of treatment. The collected root exudates over 6 h, were initially analysed for conductivity (A), K^+^‐content (B), and UV absorption spectra (190–280 nm) (C) for treatment specific effects. Values presented are means ± SE (*n* = 8, one way ANOVA, Tukey's post hoc test, *p* < 0.05 for significant differences). Different letters denote statistically significant differences among the four treatments. Control samples grown on full strength media are labelled as “C”. For comparison, tissue K^+^‐ and Ca^2+^‐concentrations from the same plants (root and shoot) are given in Figure S5, while changes in the conductivity and pH of growth media over the 10‐days treatment time are shown in Figure S6. [Color figure can be viewed at wileyonlinelibrary.com]

The next analysis addressed the effect of ‐N, ‐Ca and ‐Ca/‐N on barley root exudation and revealed a treatment specificity. The electrical conductivity data, a measure of free ions in the growth media, showed a 49% and 56% reduction under ‐N, and ‐Ca/‐N respectively, while conductivity was similar to control for the ‐Ca‐treatment (Figure 3A). The conductivity changes could not be explained by K^+^‐ions in the exudates, which only decreased by 30% for ‐N exposure, with no significant change for others (Figure 3B). Interestingly, the ‐N and the combined deprivation conditions possessed a unique signature as indicated via the UV spectra (Figure 3C). While, growth media pH gradually increased over the 10 d, conductivity varied marginally over time, with mostly overlapping effects for ‐N, and ‐Ca/‐N‐treatments (Figure S6). Further, the apparent ionic imbalance across the treatments results in a minor but uniform change (~20%) in root cell sap osmolarity for all treatments, while no differences were observed in shoot (Figure S6).

**Figure 4:**
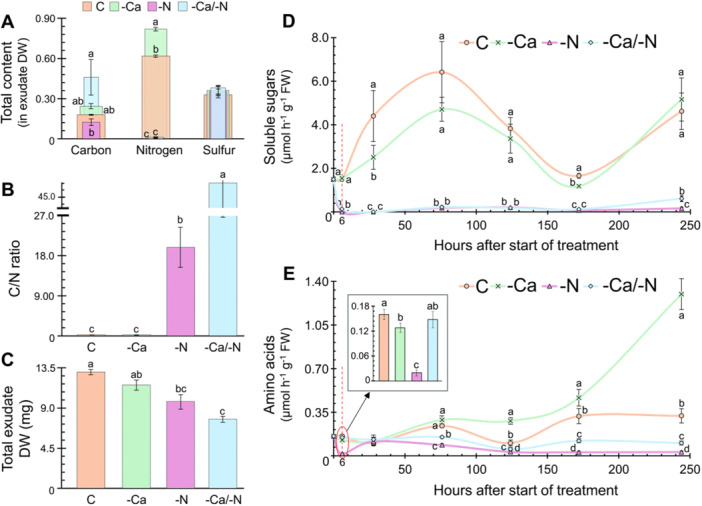
Kinetics of barley root exudation rate in terms of sugars and amino acid contents under ‐N, ‐Ca and ‐Ca/‐N. Hydroponically grown barley seedlings were analysed for root exudation. Root exudates were collected in freshly prepared nutrient media, of respective nutrient availability, on different times during the 10 days treatment period. The root exudates were collected over 6 h and the exudation rates calculated. Exudates were analysed on d 10 for total loss of C and N (A), change of exudate C/N ratio under different treatments (B), total dry weight of the lyophilised exudates (C) as well as the kinetics of exudation of sugars (D) and amino acids (E). Values presented are means ± SE (*n* = 3 for A–C, *n* = 4–30 for D, E, 2–4 independent experiment for each time point, one way ANOVA, Tukey's post hoc test, *p* < 0.05 for significant differences; Welch‐ANOVA and Games‐Howell post hoc analysis for exudation kinetics). Different letters denote statistically significant differences among the four treatments for a specific parameter (A–C) or on a specific treatment time point (D, E) (time t0 corresponds to the values in media alone). Control samples grown on full strength media are labelled as “C”. The bar chart as inset shows treatment‐specific differences in amino acid exudation rate at 6 h time point. Also, growth kinetics of plants in optimal nutrient conditions, in terms of fresh weight accumulation, during the 10 d of treatment time is presented in Figure S7. Further, a comparison of tissue sugar and amino acid contents to exudation is provided in Figure S8. [Color figure can be viewed at wileyonlinelibrary.com]

Element analysis revealed specific changes in C‐ and N‐contents of the exudates under different treatments, while S‐exudation remained unaffected (Figure 4). For example, a minor 36% increase or a 31% decrease in total C, exuded over 6 h was observed for ‐Ca and ‐N, respectively, but a 2.57‐fold increase was evident for the ‐Ca/‐N combination (Figure 4A). In contrast, the high nitrogen exudation in both control and ‐Ca treatment (32% higher than control) was reduced by 98.9% and 98% under ‐N and ‐Ca/‐N (Figure 4A). As a consequence, the C/N ratio in root exudates increased by 68.9‐ and 166‐fold relative to the control for ‐N and ‐Ca/‐N treatments, respectively (Figure 4B). The magnitude of change in C/N for both these treatments was also significantly different from each other. A similar trend existed for tissue C/N ratio, except that both treatments had indistinguishable effects in the tissue (Figure S4). A general reduction in dry weight‐related exudation was evident for both ‐N (26%) and ‐Ca/‐N‐combined treatment (40%) (Figure 4C).

Nutrient starvation of plants develops over time after start of the deficiency treatment, and exudation, being a dynamic process, changes over the treatment duration as well. Therefore, the kinetics of exudation over 10 days were recorded through quantification of soluble sugars and AAs in collected exudates. In general, exudation displayed a multiphasic pattern, with tendency to sharply increase around third day with subsequent drop between fifth and seventh day before rising again. This response was visible under all treatments for both sugars and AAs, with differences in magnitude (Figure 4D,E). This multiphasic variation in exudation, even under optimal nutrient conditions, might be linked to the growth kinetics of barley seedlings during the treatment time (Figure S7). Surprisingly, in terms of treatment duration, the differences in sugar exudation were evident within 6 h of the start (Figure 4D). Soluble sugar exudation decreased to a level close to the detection limit for ‐N and by 91% in ‐Ca/‐N within 6 h. Sugar exudation stayed low for both treatments except after d 10, when a significant difference appeared among these two, with exudation being only 4% of the control in ‐N, but 13% in ‐Ca/‐N (Figure 4D). Minor differences between control and ‐Ca treatment were visible throughout the 10 days period.

AA exudation was more dynamic, with more instances of differences among the treatments. For example, after 6 h, ‐N roots displayed reduced AA exudation (88%), while the other two treatments revealed a minor suppression (12%–20%) (Figure 4E). Except after d 5, ‐N roots consistently had lower AA exudation than ‐Ca/‐N roots. In contrast to sugars, AA exudation was consistently higher in ‐Ca compared to control from d 2 onwards, with the highest increase of 2.72‐ and 4.04‐fold after days 5 and 10, respectively (Figure 4E). Comparisons of tissue and exudate sugar/AA proportions revealed that the decreased exudation of AA occurred in parallel to root AA contents in ‐N, ‐Ca and ‐Ca/‐N, while the decrease in soluble sugar exudation tentatively correlated with soluble sugars of the shoot (Figure S8). In addition, insoluble sugars accumulated for ‐N and ‐Ca/‐N treatments in barley roots (Figure S8). Additional root exudation assays after addition of NaCl to supplement for missing Na^+^/Cl^‐^ showed a similar, prominent decrease in N‐starvation‐dependent exudation (Figure S9). An interference of excess Na^+^ or Cl^‐^ is evident in ‐Ca (after 10 days) (Figure S9). Especially for ‐Ca/‐N condition (AAs, 6 h), this interaction further intensified the N‐starvation‐dependent effect.

**Figure 5:**
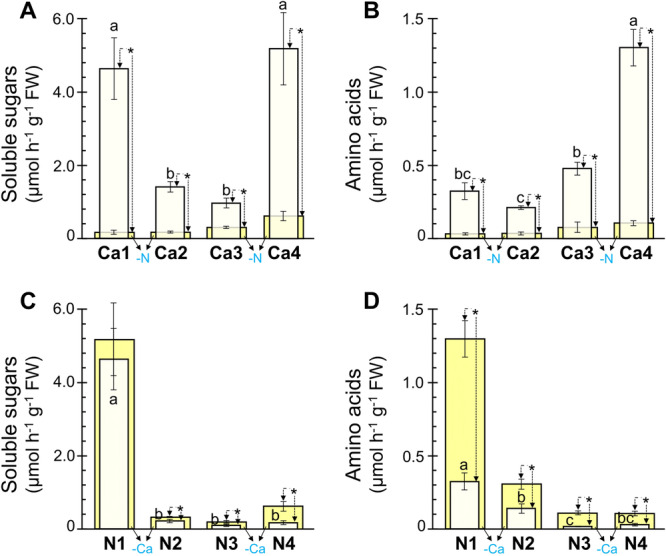
Dependence of root exudation of sugars and amino acids on external changes in Ca^2+^ or N concentration. Hydroponically grown barley seedlings were analysed for root exudation and its dependence on changes in N (N1: 3.5 mM, N2: 0.58 mM, N3: 0.016 mM, and N4: 0 mM) or Ca^2+^ (Ca1: 1 mM, Ca2: 167 µM, Ca3: 4.63 µM, and Ca4: 0 mM) concentrations in the growth media. The figure shows exudation rates for sugars (A, C) and amino acids (B, D) in dependence of Ca^2+^ availability (± N; A, B) or N availability (±Ca; C, D). Values presented are means ± SE (*n* = 4‐12, Student *t*‐test, *p* < 0.05 for significant differences). Different letters denote statistically significant differences among the four bars presenting data for Ca^2+^‐ (A, B) or NO_3_^‐^ dilution series (C, D) at optimal levels of either N or Ca^2+^, respectively. Also, significant differences from students *t*‐test are indicated by asterisks for comparison of two means ±N or ±Ca^2+^ at each dilution level (*p* < 0.01 for all these comparisons). [Color figure can be viewed at wileyonlinelibrary.com]

To further understand the combined effects of Ca^2+^‐ and N‐starvation on exudate release, exudation was analyzed 10 days after root exposure to a degressive Ca^2+^ concentration series (Ca1: 1×, 1 mM; Ca2: 1:6×, 167 µM; Ca3: 1:216x, 4.6 µM and Ca4: ‐Ca) in presence of optimal N or its complete absence and the same for a N‐dilution series ±Ca^2+^ (Figure 5). Release of soluble sugars, in contrast to AAs, significantly decreased after lowering Ca^2+^ availability to 1:6th (70%; Ca2) and 1:216th (79%; Ca3), before a full recovery was observed at ‐Ca (Ca4) (Figure 5A). On the other hand, AA exudation increased significantly (4.04‐fold of control) at ‐Ca, compared to all other Ca^2+^ dilutions (Figure 5B). For both sugars and AAs, a significant decrease in exudation rate was seen at all Ca^2+^ dilutions when N was missing (Figure 5A,B). For N‐dilution series, in general, a significant decrease in exudation rate was visible with a slight magnitude difference for sugars and AAs. For example, at 1:6th N‐dilution (N2: 0.58 mM), sugar exudation decreased by 95%, while the same was 58% for AAs (Figure 5C,D). Except for sugars at optimal N, lack of Ca^2+^ significantly increased exudation rates (Figure 5C,1D).

### 3.4 Effects of Calcium Channel Blockers on Root Exudation

**Figure 6:**
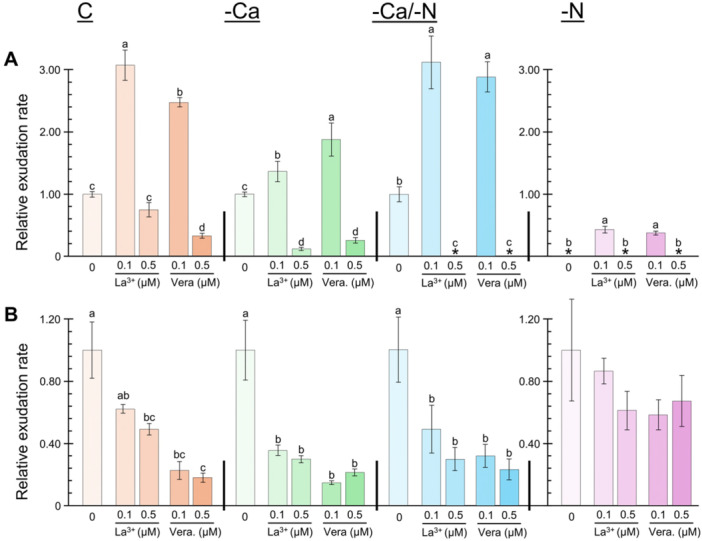
Barley root sugar exudation under ‐N, ‐Ca and ‐Ca/‐N as affected by presence of Ca^2+^ channel inhibitors. Hydroponically grown barley seedlings were analysed for root exudation after 10 days of exposure to ‐N, ‐Ca and ‐Ca/‐N in the presence of Ca^2+^‐channel inhibitors. Thus, La^3+^ (0.1, 0.5 µM) and Verapamil (Vera, 0.1, 0.5 µM) application affected sugar exudation under different nutrient deprivations after 6 h (Day 0; A) and 244 h (Day 10; B) is given. Values presented are relative exudation rates calculated for individual treatments. Thus, the exudation for different inhibitor concentrations is relative to the exudation under either control, ‐Ca, ‐N or ‐Ca/‐N without inhibitor (exception: ‐N, d 0 sugars exudation could not be detected) (means ± SE, *n* = 4–6, one way ANOVA, Tukey's post hoc test, *p* < 0.05 for significant differences). Different letters denote statistically significant differences among the five bars at a specific treatment showing the effect of the inhibitors. Control samples grown on full strength media are labelled as “C”. Asterisks mark exudation rates with severe reduction and values below the detection limit of applied methods. The selection of inhibitor concentrations used here was based on preparatory experiments where inhibitory effects were monitored over a broad range of tested inhibitor concentrations (Figure S10). [Color figure can be viewed at wileyonlinelibrary.com]

**Figure 7:**
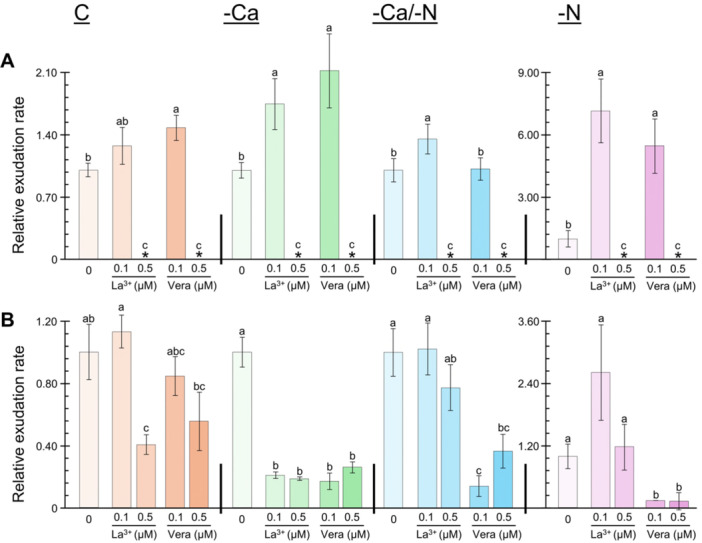
Barley root amino acid exudation under ‐N, ‐Ca and ‐Ca/‐N as affected by presence of Ca^2+^ channel inhibitors. Hydroponically grown barley seedlings were analysed for root exudation after 10 days of exposure to ‐N, ‐Ca and ‐Ca/‐N in the presence of Ca^2+^ channel inhibitors. Thus, La^3+^ (0.1, 0.5 µM) and Verapamil (Vera, 0.1, 0.5 µM) application affected amino acid exudation under different nutrient deprivations after 6 h (Days 0; A) and 244 h (Days 10; B). Values presented are relative exudation rates calculated for individual treatments. Thus, the exudation for different inhibitor concentrations is relative to the exudation under either control, ‐Ca, ‐N or ‐Ca/‐N without inhibitor (means ± SE, *n* = 4‐6, one way ANOVA, Tukey's post hoc test, *p* < 0.05 for significant differences). Different letters denote statistically significant differences among the five bars at a specific treatment showing the effect of the inhibitors. Control samples grown on full strength media are labelled as “C”. Asterisks mark exudation rates with severe reduction and values below the detection limit of applied methods. The selection of inhibitor concentrations used here was based on preparatory experiments where inhibitory effects were monitored over a broad range of tested inhibitor concentrations (Figure S10). [Color figure can be viewed at wileyonlinelibrary.com]

Evidently, Ca^2+^ deprivation with or without N‐depletion had a strong influence on root exudation. Ca^2+^ has a prominent function in cell signalling in addition to its role as structural component of cell walls. Therefore, manipulating Ca^2+^ fluxes by applying selective (verapamil) and non‐selective (Lanthanum, La^3+)^ Ca^2+^ channel blockers mostly showed overlapping effects. The differences showed a dependence on duration of nutrient starvation and inhibitor exposure time (Figures 6, 7).

For example, after a 6 h treatment and simultaneous incubation with blockers, the exudation rate was significantly higher under all treatments at 0.1 µM blocker concentration. Specifically, highest increase in soluble sugar exudation was evident under control conditions (3.07‐fold: 0.1 µM La, 2.47‐fold: 0.1 µM verapamil) and ‐Ca/‐N (3.12‐fold: 0.1 µM La, 2.89‐fold: 0.1 µM verapamil) (Figure 6A). At this time point, exposure to 0.5 µM blocker concentration led to substantial reduction of exudation for all treatments, with the magnitude being highest at both treatments involving Ca^2+^ depletion, i.e. 88% for La^3+^, and 75% for verapamil under ‐Ca, while values were below detection limit for ‐Ca/‐N (Figure 6A). Sugar exudation was also below detection limit at higher blocker concentrations for ‐N. In comparison, the AAs exudation displayed very similar bidirectional behaviour at 0.1 and 0.5 µM blocker concentrations. The increase in AAs exudation at 0.1 µM was significantly different for both blockers for ‐Ca and ‐N, while at 0.5 µM La^3+^/verapamil, for all treatments exudation values were too low to be detected (Figure 7A). Interestingly, the increase for ‐N was substantially higher than for all other treatments, that is, 7.15‐fold and 5.47‐fold at 0.1 µM La^3+^ and verapamil, respectively (Figure 7A).

After 10 d of treatment and simultaneous exposure to Ca^2+^ channel blockers, the inhibitor effect was quite contrasting, especially at lower concentrations. Thus, for all treatments, but ‐N, both blocker concentrations reduced soluble sugar and AA exudation (Figures 6B, 7B). Generally, verapamil had stronger effects after 10 d than La^3+^. External lack of Ca^2+^ availability intensified the blocker effect causing higher suppression of exudation at all blocker concentrations (65%–85%) for both sugars and AAs (Figures 6B, 7B). As mentioned above, sugar exudation in ‐N was not significantly influenced by Ca^2+^ channel blockers, while only verapamil significantly affected AA release (decrease by 85, and 87% at 0.1, 0.5 µM verapamil, respectively), tentatively indicating regulation involving voltage‐gated Ca^2+^ channels (Figures 6B, 7B). The Ca^2+^ channel blocker effect was unique in ‐Ca/‐N, where sugar exudation was strongly suppressed by both blockers with concentration specificity, while only verapamil application reduced AA exudation (63%–86%; Figures 6B, 7B).

### 3.5 Identification of Exudated Metabolites by Metabolite Profiling

**Figure 8:**
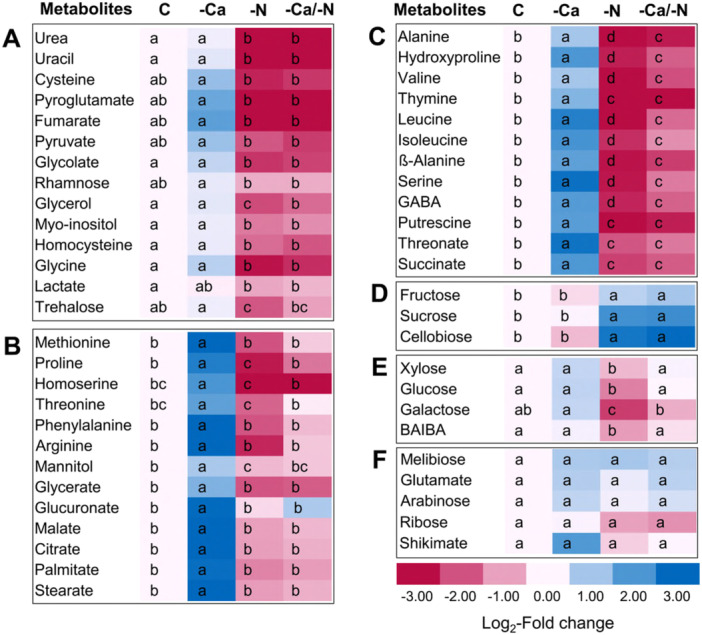
Metabolic signature of root exudates from barley grown in ‐N, ‐Ca, ‐Ca/‐N). Hydroponically grown barley seedlings were analysed for root exudation, after 10 days of exposure, through metabolite profiling of freeze‐dried exudate samples. The GC‐MS‐based analysis is described in Materials and Methods section. The identified metabolites were grouped into six major categories based on the treatment specificity of the relative amounts and statistically significant differences among the treatments. Thus, the heatmap presented here shows log_2_ fold changes for relative metabolite content under different treatments compared to the control (*n* = 10 from five independent plant cultures, one way ANOVA, Tukey's post hoc test, *p* < 0.05 for significant differences). Group (A) comprises metabolites with ‐N‐dependent decrease, (B) ‐Ca‐specific increase, (C) significant deviation from control for all treatments, (D) ‐N‐dependent increase or ‐N‐specific decrease (E), and no treatment effects (F). Principal component analysis for the root exudate metabolites, carried out using online tool *MetaboAnalyst* (v6.0; MetaboAnalyst), is given in Figure S11. Further, the detailed relative metabolite analysis under these four conditions is presented in Table S1. [Color figure can be viewed at wileyonlinelibrary.com]

Quantifying the release of total sugars and AAs enabled insights into the conditional dynamics of exudation. Next, we aimed at identifying specific metabolites that were either increased or decreased in exudation upon the different treatments (Figure 8, Table S1, Figure S11). GC‐MS identified 51 compounds in the exudates. The major fraction comprised primary metabolites with changes in exudation after 10 d of treatment. A set of 15 compounds formed the largest category that showed a significantly decreased exudation under ‐N, and ‐Ca/‐N, as compared to control and ‐Ca (Figure 8A). Some AAs (Gly, Cys, Homo Cys etc), OAs (fumarate, lactate, pyruvate, glycolate) and sugars (rhamnose, trehalose, ribose) followed this exudation pattern. Exudation of urea (‐4.35, ‐3.91 log_2_ fold) and uracil (‐6.18, ‐5.98 log_2_ fold) was significantly reduced under ‐N and ‐Ca/‐N (Figure 8A). Another set of 13 metabolites with a significant increase (~2.97 log_2_ fold average increase) under ‐Ca‐treatment included the AAs Met, Pro, HomoSer, Thr, Phe and Arg along with the OAs glucuronate, malate, citrate and glycerate and the fatty acids palmitate and stearate (Figure 8B). Most pronounced increases under ‐Ca treatment were detected for Phe (6.17 log_2_ fold), Met (4.29 log_2_ fold), and malate (3.91 log_2_ fold) (Figure 8A, Table S1).

Exudation of several metabolites strongly increased under ‐Ca with parallel significant reduction in both ‐N and ‐Ca/‐N (Figure 8C). For example, while exudation of thymine and putrescine increased under ‐Ca by 1.36 and 1.85 log_2_ fold, respectively, the same was reduced under ‐N (‐4.50, ‐3.64 log_2_ fold) and ‐Ca/‐N (‐4.28, ‐2.73 log_2_ fold) (Figure 8C, Table S1). Group C revealed the most diverse response in magnitude and significance levels.

While N‐containing metabolites are part of Figure 8A–C with the general trend towards reduced exudation under ‐N and ‐Ca/‐N, specifically the exudation of the three sugars fructose, sucrose and cellobiose increased under N‐starvation (Figure 8D). Thus, fructose (0.76, 0.99 log_2_ fold), sucrose (2.11, 2.19 log_2_ fold), and cellobiose (2.69, 2.95 log_2_ fold) were unique in their increased root exudation for both ‐N and ‐Ca/‐N‐ (Figure 8D). Few other sugars like xylose, glucose, and galactose showed ‐N specificity and were reduced significantly in exudation under ‐N (Figure 8E). Interestingly, melibiose, glutamate, arabinose, ribose and shikimate largely showed equal root exudation under all nutritional conditions and were not influenced by N, or Ca‐nutritional status (Figure 8F).

**Figure 9:**
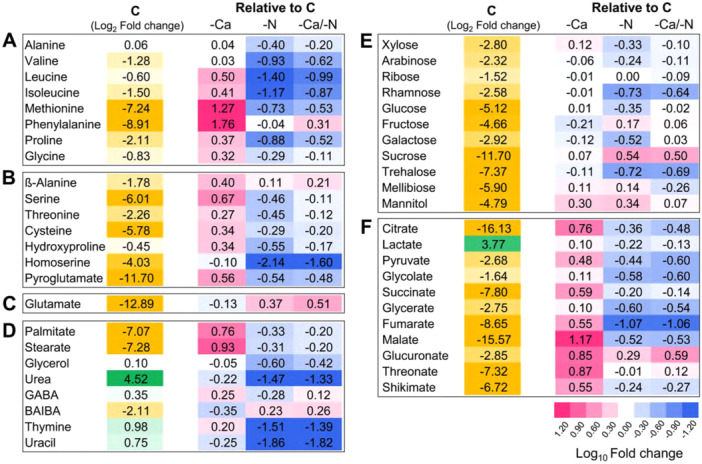
Comparison of metabolite pools in total root extract relative to root exudates of barley grown in ‐N, ‐Ca and ‐Ca/‐N. The relative metabolite contents in root tissue and root exudate are presented in two different formats. First, root exudate metabolites are given for control samples, as log_2_ fold change compared to amounts in root tissue (shades of golden yellow, green), for example, urea in exudate was 4.52 log_2_ fold (22.9‐fold) more represented than in root extract. Second, the changes in these metabolite proportions (increased or decreased) in exudates for the three treatments, that is, ‐Ca, ‐N or ‐Ca/‐N are given as log_10_ fold‐changes as compared to “C” in the heat maps (check relevant scale). The different panels represent categories (A) non‐polar amino acids, (B) polar amino acids, (C) charged amino acids, (D) other metabolites, (E) sugar and sugar‐derivatives, (F) organic acids. Principal component analysis for the root (and shoot) metabolites, carried out using online tool *MetaboAnalyst* (v5.0; MetaboAnalyst), is given in Figure S12. Further, the raw relative metabolite contents measured in these four conditions for roots and shoots are presented in Tables S2, S3 (*n* = 6 for root and shoot tissue metabolites). [Color figure can be viewed at wileyonlinelibrary.com]

Further, root tissue metabolite levels were compared to those in collected root exudates and proportions calculated to demonstrate how the metabolite specific flux might differ for the conditions under consideration (Figure 9, Figure S12). In general, among the six categories of metabolites presented, it is apparent that the proportions of metabolites in root exudates to those in root tissue given under optimal conditions, (control) drastically differed for different metabolites and treatments (Figure 9A–F). A strong concentration gradient might exist from roots to rhizosphere for most metabolites, as is visible after 6 h of exudation (Figure 9A–F, control conditions). Interestingly, few metabolites showed higher content in exudates compared to root tissue within 6 h under optimal conditions, e.g. urea (4.52 log_2_ fold), lactate (3.77 log_2_ fold), thymine (0.98 log_2_ fold) and uracil (0.75 log_2_ fold) (Figure 9D,F).

In general, most metabolites increased in exudate fraction under ‐Ca and decreased under ‐N, while often ‐Ca/‐N showed changes parallel to ‐N conditions with differences in magnitude (Figure 9). For example, multiple non‐polar AAs strongly increased in proportions in root exudates under ‐Ca, e.g. Met (1.27 log_10_ fold), Phe (1.76 log_10_ fold), and Pro (0.37 log_10_ fold), along with several polar AAs like Ser, Thr, and PyroGlu etc. (Figure 9A,B). Importantly, N‐assimilation metabolites with function in nitrate sensing, and the charged AA Glu increased (0.37, and 0.51 log_10_ fold) in proportions under ‐N, and ‐Ca/‐N treatments, respectively (Figure 9C). Also, fatty acids palmitate and stearate, decreased for ‐N, and ‐Ca/‐N treatments along with glycerol, urea, thymine and uracil (Figure 9D). Further, comparison of the response of several sugars indicated a general decrease for all treatments, while some showed ‐N and ‐Ca/‐N treatment‐specific increases, for example, sucrose (0.54, and 0.50 log_10_ fold) (Figure 9E).

## 4 Discussion

Evidence in support of an important role of root exudation in plant acclimation to the environment is growing rapidly (Chai and Schachtman 2022). Regulation of root exudation is highly dependent on rhizospheric chemistry, besides physical and biological features (Badri and Vivanco 2009). This study shows that barley plants rapidly adjust root exudation upon ‐N or ‐Ca starvation, the direction of which is largely contrasting. A combination of these conditions, although unique in several aspects, aligns closely with N‐starvation, but also highlights the potential regulatory role for Ca^2+^‐signalling in root exudation. The findings of this study can be evaluated in context of three major questions: How does the recorded exudation profile fit into management of actual plant N status, and thus plant growth? Is Ca^2+^‐dependent regulation of exudation multifaceted? What is the role of the differentially exudated metabolite in the nutrient starvation response?

### 4.1 Perceived and Realised N‐Starvation Controls Root Exudation?

It has been reported that 3 d N‐starvation suppresses root exudation of AAs in barley (Carvalhais et al. 2011). However, in the current setup, this suppression is already apparent after 6 h of perceived N‐limitation in the growth media and is not limited to AAs but includes sugars as well (Figure 4). Further, this AA exudation appears to be Ca^2+^‐dependent, as visible in the contrasting exudation for ‐Ca/‐N at 6 h compared to ‐N, as well as in the Ca^2+^ channel inhibitor treatments (Figures 4E, 6A, 7A).

It can be hypothesised that this effect is related to an early N‐sensing event and potentially regulated by rapid inhibition of involved transporters. The dual‐affinity NO_3_^‐^ transporter **N**it**R**ate **T**ransporter 1.1 (NRT1.1) is known to trigger Ca^2+^ signalling (Adavi and Sathee 2024), which regulates the **P**rimary **N**itrate **R**esponse (PNR). PNR comprises of genome‐wide expression change targeted at plasticity of N‐assimilation and root growth (Ho et al. 2009; Maghiaoui et al. 2020; Medici and Krouk 2014). However, the molecular mechanisms sensing the absence of nitrate are unknown (Ruffel et al. 2025). Although, a nitrate‐independent **N**itrate‐**S**tarvation **R**esponse (NSR) has been described in literature and is induced by complete lack of nitrate (Menz et al. 2016). The rapidity of NSR compared to PNR is considered moderate (Ruffel et al. 2025). However, diverse N‐starvation studies in rice, legumes (e.g., *Medicago truncatula* Gaertn.) and different barley genotypes have shown transcriptional changes for different classes of metabolite transporters within 24 h of external N starvation (Table S4) (Cai et al. 2012; Decouard et al. 2022; Quan et al. 2016; Ruffel et al. 2008; Yang et al. 2015). Further, despite additional controls included to address ionic balance (Figure S9), the removal of specific salts (NaNO₃ and CaCl₂) could potentially alter both ionic identity and valence in the external medium. The current controls cannot fully exclude that the rapid adjustment to early exudation is due to ionic composition or membrane potential changes rather than nutrient‐specific signalling alone. Also, although it is shown that the pH range remained within agronomically acceptable limits, even small and rapid shifts in proton availability may influence transporter activity and metabolite fluxes and should be considered when interpreting short‐term exudation responses.

Interestingly, in Tibetan wild barley, transcripts for several AA or sugar transporters were deregulated within 6 h of N starvation (Table S4) (Quan et al. 2016). The transcriptional changes indicate a rapid metabolic adjustment to the external N‐status that could play a role in the changed root exudation profile under ‐N and ‐Ca/‐N. This could further contribute to cellular N economy. Further work is required to establish the link between the N‐starvation response and exudation control, as well as potential involvement of Ca^2+^ signalling in regulating the metabolite transporters involved.

N starvation is rapidly perceived in form of dropping nitrate content in the plant. Possibly, this mechanism leads to recalibration of the cellular response, resulting in a minor increase of root AA exudation, between 24 and 48 h followed by a consistent decrease (Figure 4). The change in internal nitrate correlates with suppressed NR activity (Figure 2) (Adavi and Sathee 2024; Chen et al. 2022). Recent findings implicate **N**odulin Inception (NIN)‐**L**ike **P**rotein 7 (NPL7) as an evolutionarily conserved intracellular nitrate sensor, besides being a transcription factor in control of PNR under a broad range of nitrate concentrations (Liu et al. 2022). The change in internal nitrate perturbs NPL7 phosphorylation which in turn modifies PNR and N‐homoeostasis‐related processes (Menz et al. 2016), potentially modifying root exudation profile. Altered activities of several metabolite transporters, which are part of the PNR (Table S4) (Canales et al. 2014), could cause the observed root exudation pattern.

Interestingly, NPL7 also coordinates N and C metabolic pathways to control provisions of reducing power and C‐skeletons for N assimilation in cooperation with NPL2 (Ariga et al. 2022; Durand et al. 2023). A ‐N‐induced metabolic shift in C‐metabolism is visible in reduced soluble or increased insoluble tissue sugars in both ‐N, and ‐Ca/‐N conditions, and is also reflected in tissue C/N ratio (Figures S4, S8). Ca^2+^ appears to play a significant role in this regulation as well, as Ca^2+^ deprivation for 10 d strongly increased the C/N ratio in root exudates for ‐Ca/‐N conditions compared to ‐N (Figure 4B). An enhanced PNR has been reported in plants that are exposed to Ca^2+^‐depleted media, especially under low nitrate (Ho et al. 2009). The current study highlights the need for understanding root exudation as potential part of PNR or NSR and for identifying specific targets of Ca^2+^ signalling among transporter proteins and their regulators.

### 4.2 Ca^2+^ as Nutrient and/or Signal in Regulation of Root Exudation

A step wise reduction in AA exudation after 10 d exposure to a N‐dilution series (Figure 5) strongly indicates a calibrated cellular response, potentially through the internal nitrate sensor like NPL7. The presence of optimal external Ca^2+^ appears to be a critical factor in this regulation. Further, while Ca‐starvation alone dramatically increased AA exudation, but not that of sugars, a strong decline in both indicates a dominance of N‐starvation responses in ‐Ca/‐N (Figures 4, 5). Influence of Ca^2+^ on membrane permeability is known, either as a structural component (Jing et al. 2024; Thor 2019) or through the Ca^2+^ fluxes across the membrane (Wdowiak et al. 2024), altering transporters function (Dong et al. 2022a). While the first process might result in a “leaky membrane” phenotype, involvement of the second would better explain specific effects on AAs exudation and ion loss (Figure 3).

Support for this hypothesis is provided by Ca^2+^ channel blocker data. On the one hand sugar exudation is further suppressed for both blocker types under ‐Ca/‐N treatment (same for C or ‐Ca), while AA exudation is selectively reduced only by blocking voltage‐gated Ca^2+^ channels, using micromolar range verapamil, under both ‐N and ‐Ca/‐N treatments (Figures 6B, 7B) (Demidchik et al. 2002). After 6 h of treatment, a 0.5 µM La^3+^/verapamil exposure like response, i.e. intensified reduction of the N‐dependent AA‐exudation, was observed upon NaCl application (Figure S9). This points to another critical factor in the modification of membrane potential, i.e. ionic equilibrium. It needs to be further evaluated, especially in context of interference by other essential ions.

More importantly, the highlighted dominance of N‐starvation response, when comparing ‐Ca and ‐Ca/‐N conditions, might be a result of a broader metabolic rearrangement. Multiple factors point in this direction, in particular the lowered AA biosynthesis (depleted pool in roots, Figure 8B) (Chen et al. 2022) and the repression of energy‐intensive processes (Durand et al. 2023), which is also reflected in increased tissue C/N ratio, especially in roots and increased tissue insoluble sugars (Figures S4, S8). Vesicular traffic, an energy intensive process (Dettmer et al. 2006), is required for membrane remodelling and transporter protein turnover and may be significantly impeded under N‐starvation leading to changes in membrane behaviour (Gao and Chao 2022). It has been shown that eukaryotic cells respond to amino acid depletion by increasing membrane protein turnover through TOR‐kinase‐associated starvation signalling (Jones et al. 2012; McLoughlin et al. 2018). This metabolic change could further suppress root exudation. Interestingly, membrane trafficking is regulated by annexins in a strongly Ca^2+^‐dependent manner (Konopka‐Postupolska and Clark 2017), perhaps resulting in the “leaky phenotype” in terms of root exudation in ‐Ca. However, N‐starvation overrides these strong effects, and the remaining Ca^2+^ effect must be tightly controlled, possibly by NSR‐ or PNR‐dependent mechanisms, as seen in ‐Ca/‐N.

The Ca^2+^ influence is subtle but visible in the data discussed above. Further clarity is provided in N‐dilution series, where Ca^2+^ starvation led to increased AA exudation, but was most prominent only under optimal N (Figure 5D). The processes discussed in this section are highly regulated, especially via redox and hormonal signalling, which would require further in‐depth analysis.

### 4.3 Root Exudate Metabolic Pattern May Indicate Treatment‐Specific Microbe Recruitment

Role of specific transporters for exudation of specific metabolites has been proven pharmacologically (Loyola‐Vargas et al. 2007). The specificity in the regulation of root exudation indicates an important targeted role for exuded metabolites, coupled to the source‐sink relations. Further, it has been documented in barley cultivars that genotype‐specific exudation results in differential microbial recruitment (Pacheco‐Moreno et al. 2024). In the current study, despite a pronounced downregulation of exudation under ‐N and ‐Ca/‐N conditions, exudation of fructose, sucrose and cellobiose was promoted (Figure 8). Fructose and sucrose have been identified as chemoattractants and mediators of microbial motility, chemotaxis, and biofilm formation, especially plant growth promoting *Bacillus* species (Feng et al. 2018; Tian et al. 2021). Specific strains of *B. subtilis* function as diazotrophs, contributing towards N_2_‐fixation and providing a N‐source for plants under N‐deprivation, as reported in maize (Kuan et al. 2016; Singh and Shyu 2024). On the other hand, cellobiose exudation is an indicator of cell wall remodelling under N‐deprivation, as has been reported in wheat (Meychik et al. 2017). In addition, microbial utilisation of cellobiose from root exudates has a strong positive correlation to their plant growth promoting ability (Shi et al. 2022). A strict metabolic control of exudation in N‐deprived barley seedlings is vividly visible in the suppressed exudation of easily diffusible molecules like urea, glycerol, redox‐linked cysteine and Gly or energy metabolites like glucose, glycerol, fumarate, succinate and pyruvate.

In addition, barley plants under Ca^2+^ deprivation exuded higher amounts of malate and citrate (Figure 8), potentially attempting Ca^2+^ chelation in the rhizosphere and improving nutrient availability, along with their role in pH homoeostasis (Tahjib‐Ul‐Arif et al. 2021). Further, besides being a rich N and C source for **P**lant **G**rowth **P**romoting **R**hizobacteria (PGPR), specificity in microbe recruitment has also been identified for different exudated AAs. For example, *Pseudomonas aeruginosa* strains are attracted by Ala, Arg, Met and Pro (Taguchi et al. 1997), *P. fluorescens* Pf0‐1 by a wide range of AAs (Oku et al. 2012), *P. putida* KT2440R by Ser, Phe, Met and Pro (Corral‐Lugo et al. 2016), *Azorhizobium caulinodans* ORS571 by Arg, and AA‐derived putrescine in some Pseudomonas strains. Homoserine in exudates acts as a critical quorum sensing molecule for PGPR like *Aeromonas* spp. (Nawaz et al. 2020). Recruited microbes could play different roles in plant growth homoeostasis in Ca^2+^ deprived state. Also, ‐Ca‐induced higher exudation of palmitate and stearate might indicate, Ca^2+^‐deprivation induced membrane lipid re‐profiling as reported in Arabidopsis roots (Zhang et al. 2018).

## 5 Conclusions

Barley plants exposed to N‐ and Ca^2+^‐starvation showed contrasting root exudation patterns. In comparison, plants exposed to their combination highlighted prominence of N‐starvation response in the control of metabolite exudation aimed at resource preservation. A rapid suppression of amino acid exudation within 6 h in ‐N appears to be Ca^2+^‐dependent, as the effect disappeared in ‐Ca/‐N. The kinetics of exudation over 10 days indicated a potential dependence on internal nitrate concentrations in ‐N and ‐Ca/‐N. In contrast, rapid external nitrate sensing must be required initially. Involvement of Ca^2+^ signalling is revealed through Ca^2+^ flux inhibitors, however acting downstream to plant N‐starvation with direct or indirect impact. Further, targeted exudation of fructose and sucrose under N‐starvation points to plant efforts in microbial recruitment, especially focused at improving N‐availability. Further research should explore the actual impact on the microbiome as well as on plant growth phenotype of a successful microbial recruitment under such combinations of nutrient limitations. Also, characterisation of discussed potential transporters will be valuable.

## Conflicts of Interest

The authors declare no conflicts of interest.

## Supporting information

Supporting File 1

Supporting File 2

Supporting File 3

Supporting File 4

Supporting File 5
