# Supplementary material for: Calcium and Nitrogen Availability Controls Root Exudation in Hydroponically Cultured Barley
Source: Plant Cell Environ. 2026 Apr 14;49(8):5210–27. doi: 10.1111/pce.70528 (PMC13353552; doi:10.1111/pce.70528)
Supplement: Supplementary file 1 — Supporting File 1 [file PCE-49-5210-s001.pdf]

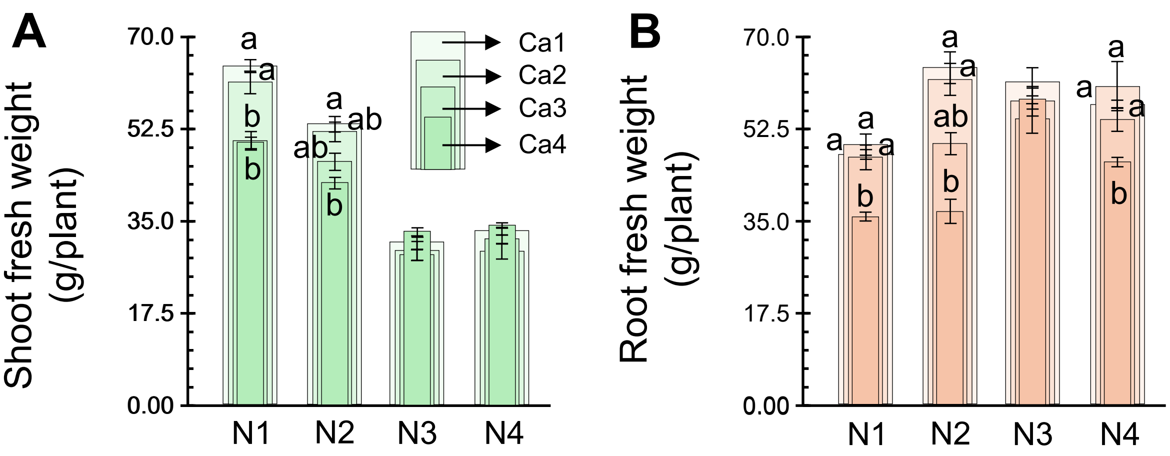

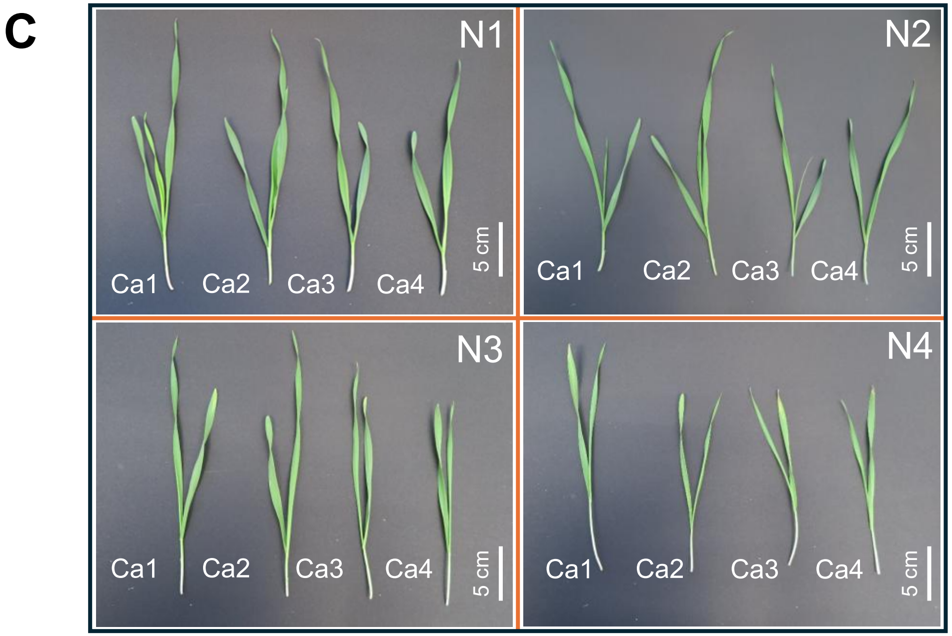

Figure S1: Growth of barley seedlings in a wide range of N, and Ca-nutritional status. Barley seedlings (*Hordeum vulgare* L. cv. SU MIDNIGHT; winter barley) were grown in hydroponics with an array of N (N1: 3.5, N2: 0.58, N3: 0.016, and N4: 0 mM), and Ca (Ca1: 1, Ca2: 0.0625, Ca3: 0.005, and Ca4: 0 mM) concentrations. Shoot (**A**) and Root (**B**) fresh weight was measured after 10 d of seedling growth on the varied nutritional media (Values are means±SE; n = 19 for all except N1Ca1 (C), N1Ca4 (-Ca), N4Ca1 (-N), N4Ca4 (-Ca/-N) where n = 100-130, one way ANOVA, Tukey's post hoc test, $p<0.05$ for significant differences). Different letters denote statistical differences between four means for each N-level individually. Plant phenotype was recorded after 10 d as well (Shoot growth phenotype; **C**). Seeds were germinated on vermiculite and seedlings grown on normal growth media before transfer to treatment conditions at 8 d age.

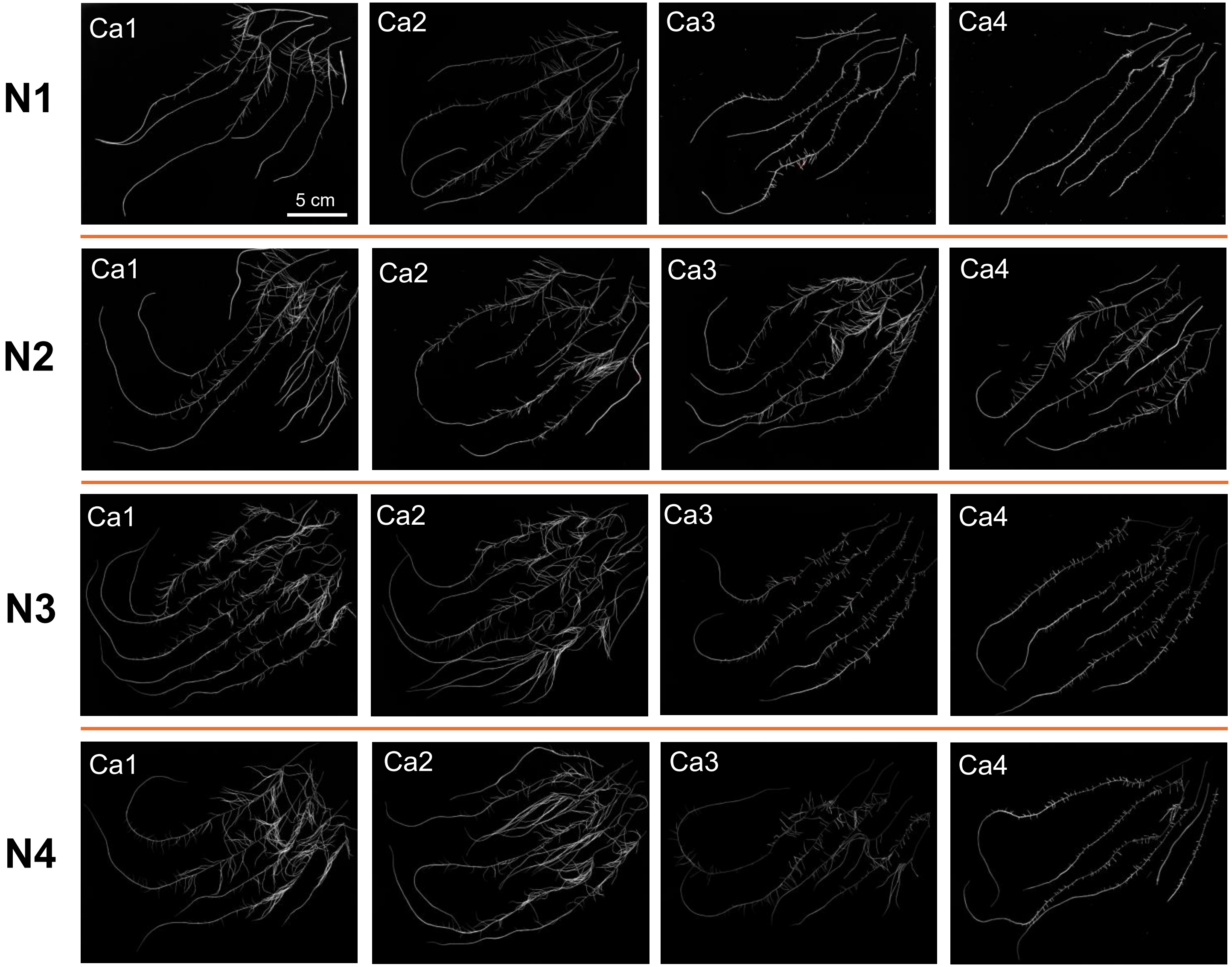

Figure S2: Representative root growth phenotype of barley seedlings grown on a wide range of N, and Ca-nutritional conditions. Barley seedlings (*Hordeum vulgare* L. cv. SU MIDNIGHT; winter barley) were grown in hydroponics with a array of N (N1: 3.5, N2: 0.58, N3: 0.016, and N4: 0 mM), and Ca (Ca1: 1, Ca2: 0.0625, Ca3: 0.005, and Ca4: 0 mM) concentrations. Root growth phenotype is shown after 10 d of growth on the treatment conditions. Seeds were germinated on vermiculite and seedlings grown on normal growth media before transfer to treatment conditions at 8 d age.

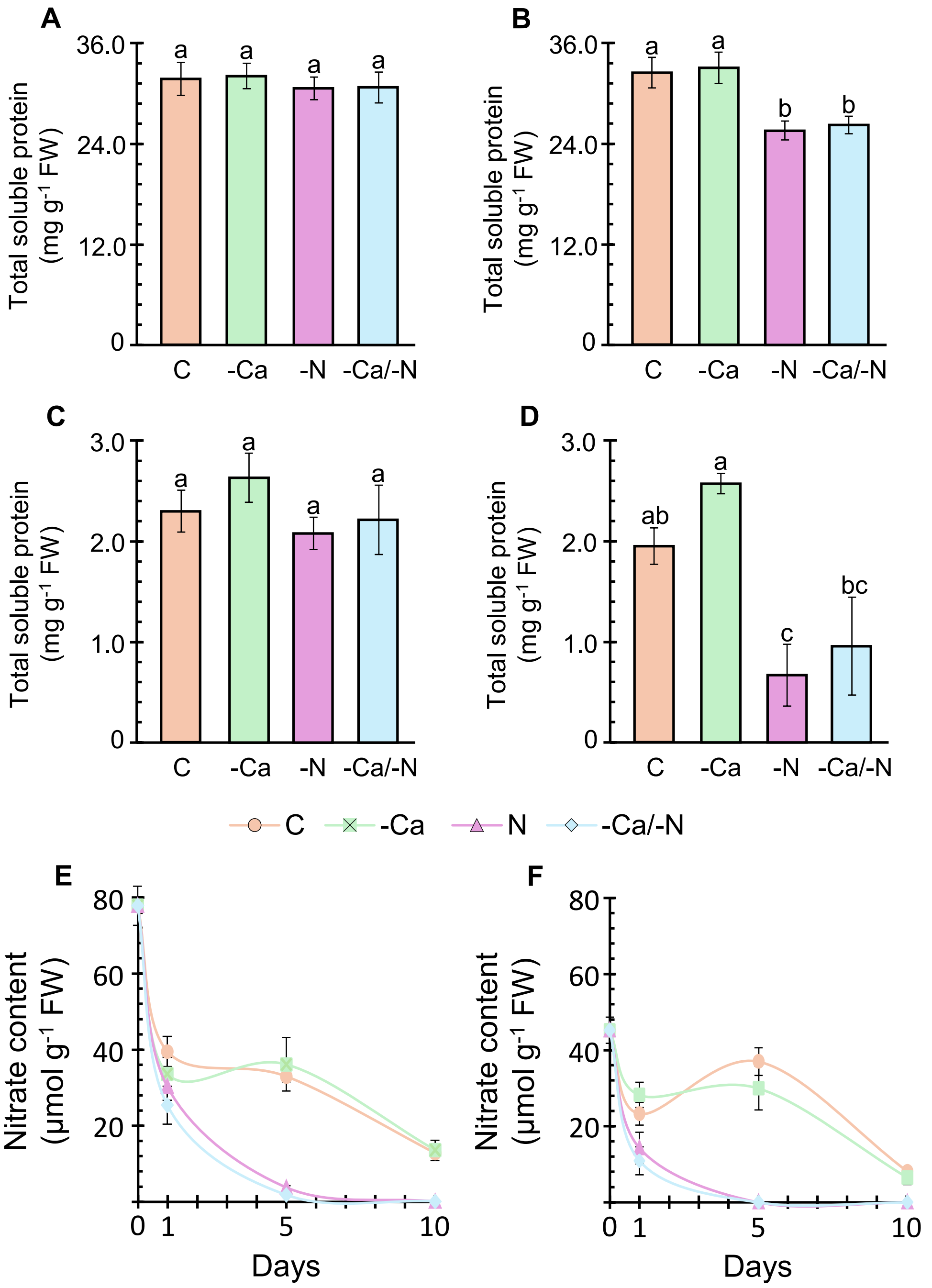

Figure S3: Effects of -N, -Ca and -Ca/-N on soluble protein and nitrate content over the treatment period. Barley seedlings (*Hordeum vulgare* L. cv. SU MIDNIGHT; winter barley) were grown in hydroponics with -N, -Ca and -Ca/-N for 10 d. Seedlings were germinated on vermiculite and grown on normal growth media before transfer to treatment conditions at 8 d age. On d 3 (**A**, **C**), and d 10 (**B**, **D**) of treatment plant shoot (**A**, **B**) and roots (**C**, **D**) were harvested and analysed for total soluble protein content. Values presented are means±SE (n = 3, 3 independent experiments, one way ANOVA, Tukey's post hoc test, $p<0.05$ for significant differences). Different letters denote statistical differences among the four treatments. Reference samples grown on full strength media are labelled as "C". Shoot (**E**) and root tissue (**F**) nitrate content measured over the 10 d treatment period (means±SE, n = 3) (time t0 corresponds to the values in media alone).

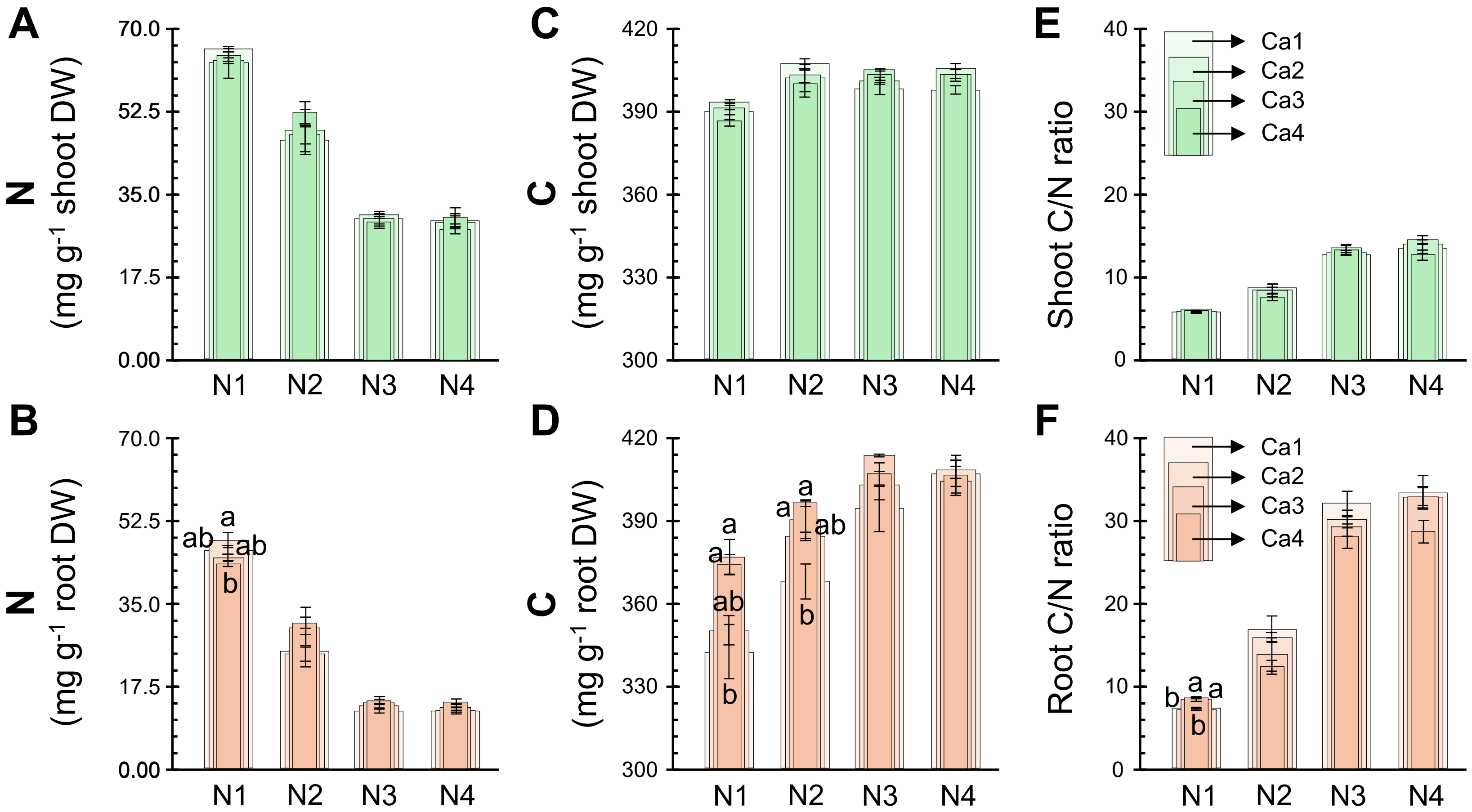

Figure S4: C- and N-contents of barley seedlings grown on varied N-, and Ca-nutritional status. Barley seedlings (*Hordeum vulgare* L. cv. SU MIDNIGHT; winter barley) were grown in hydroponics with an array of N (N1: 3.5, N2: 0.58, N3: 0.016, and N4: 0 mM), and Ca (Ca1: 1, Ca2: 0.0625, Ca3: 0.005, and Ca4: 0 mM) concentrations. Seeds were germinated on vermiculite and seedlings grown on normal growth media before transfer to treatment conditions at 8 d age. After 10 d of growth with the mentioned nutrient supply, N (**A**, **B**), and C (**C, D**)-status of shoot and root, respectively, was carried out in freeze dried samples using Unicube (Elementar, Langenselbold, Germany) along with C/N ratio (**E, F**). Sulfanilamide ($C_6H_8N_2O_2S$) was used as the reference compound. Values are means±SE (n = 4, one way ANOVA, Tukey's post hoc test, $p<0.05$ for significant differences). Different letters denote statistical differences between four means for each N-level individually.

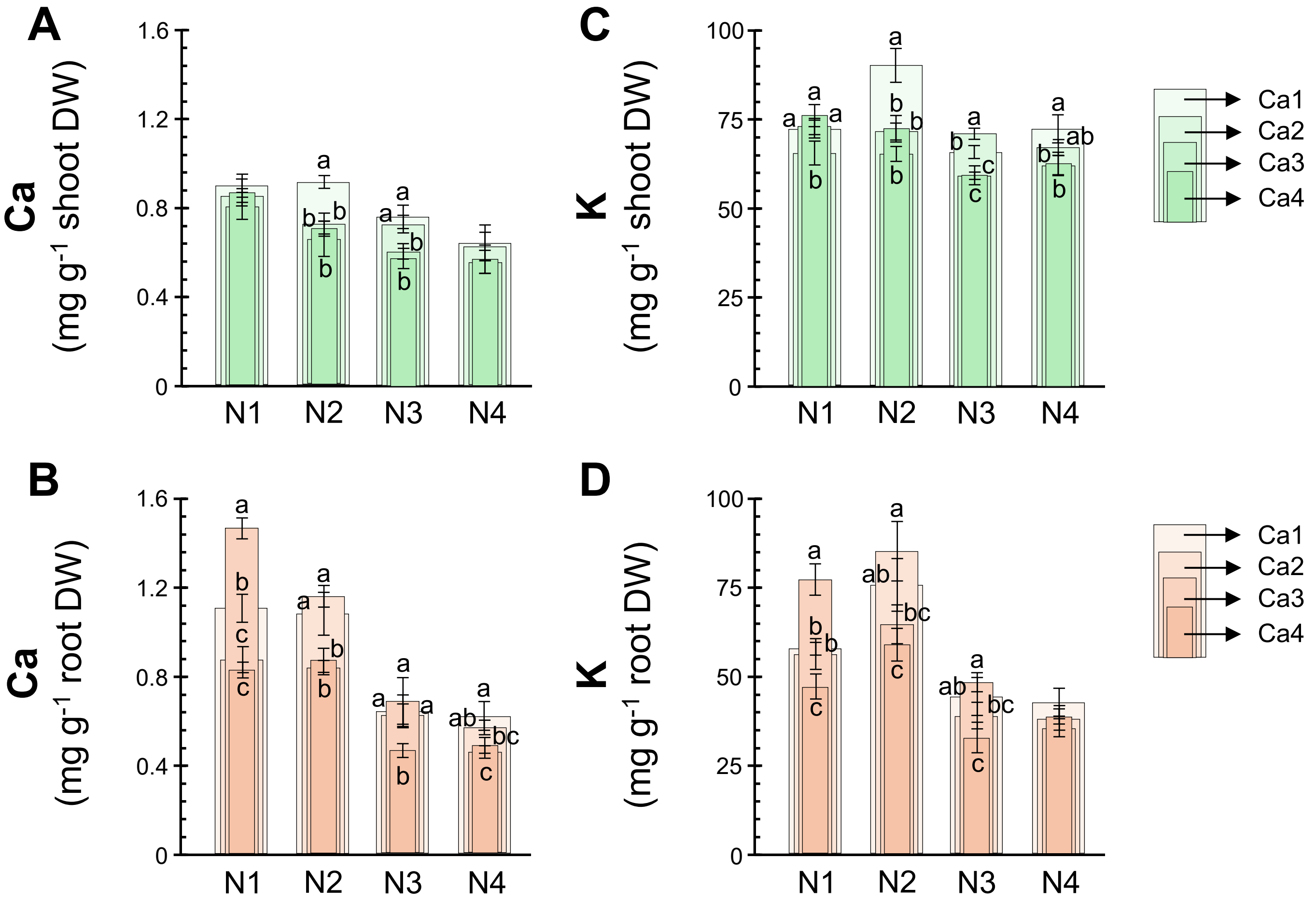

Figure S5: K, Ca-analysis in barley seedlings grown on varied N, and Ca-nutritional status. Barley seedlings (*Hordeum vulgare* L. cv. SU MIDNIGHT; winter barley) were grown in hydroponics with an array of N (N1: 3.5, N2: 0.58, N3: 0.016, and N4: 0 mM), and Ca (Ca1: 1, Ca2: 0.0625, Ca3: 0.005, and Ca4: 0 mM) concentrations. Seeds were germinated on vermiculite and seedlings grown on normal growth media before transfer to treatment conditions at 8 d age. After 10 d of growth on the mentioned nutrient supply, Ca-status of root and shoot was carried out in freeze dried samples using flame photometer (Model 410; Sherwood Scientific Ltd., UK). Samples were acid digested as explained in the MM section. K and Ca standard (1000 ppm Ca, Sherwood Scientific Ltd., UK) was used to calibrate the machine (0-10 ppm) for reference values. Values presented are means±SE ($n = 4$ for Ca, 3 for K, one way ANOVA, Tukey's post hoc test, $p<0.05$ for significant differences). Different letters denote statistical differences between four means for each N-level individually.

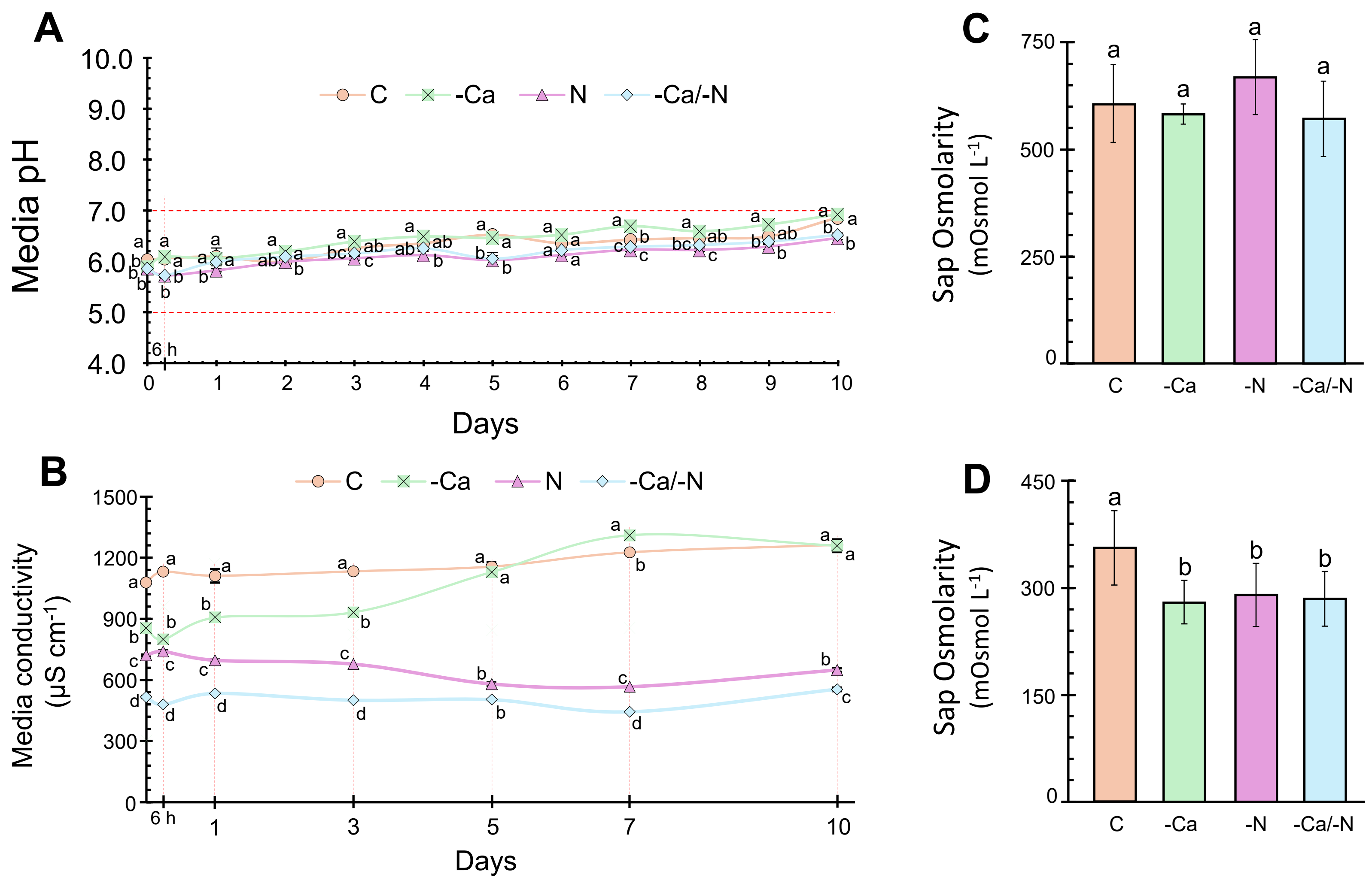

Figure S6: Response of growth media pH and conductivity under –Ca, -N and –Ca/-N conditions over the treatment period. Barley seedlings (*Hordeum vulgare* L. cv. SU MIDNIGHT; winter barley) were grown in hydroponics with –N, -Ca and –Ca/-N for 10 d. During the 10 d of treatment, changes in growth media pH (**A**) and conductivity (**B**) are given. Sap osmolarity was also quantified in shoot (**C)** and root **(D**). Values presented are means±SE (n = 3, one way ANOVA, Tukey's post hoc test, $p<0.05$ for significant differences). Different letters denote statistical differences among the four treatments at a specific treatment time point (time t0 corresponds to the values in media alone).

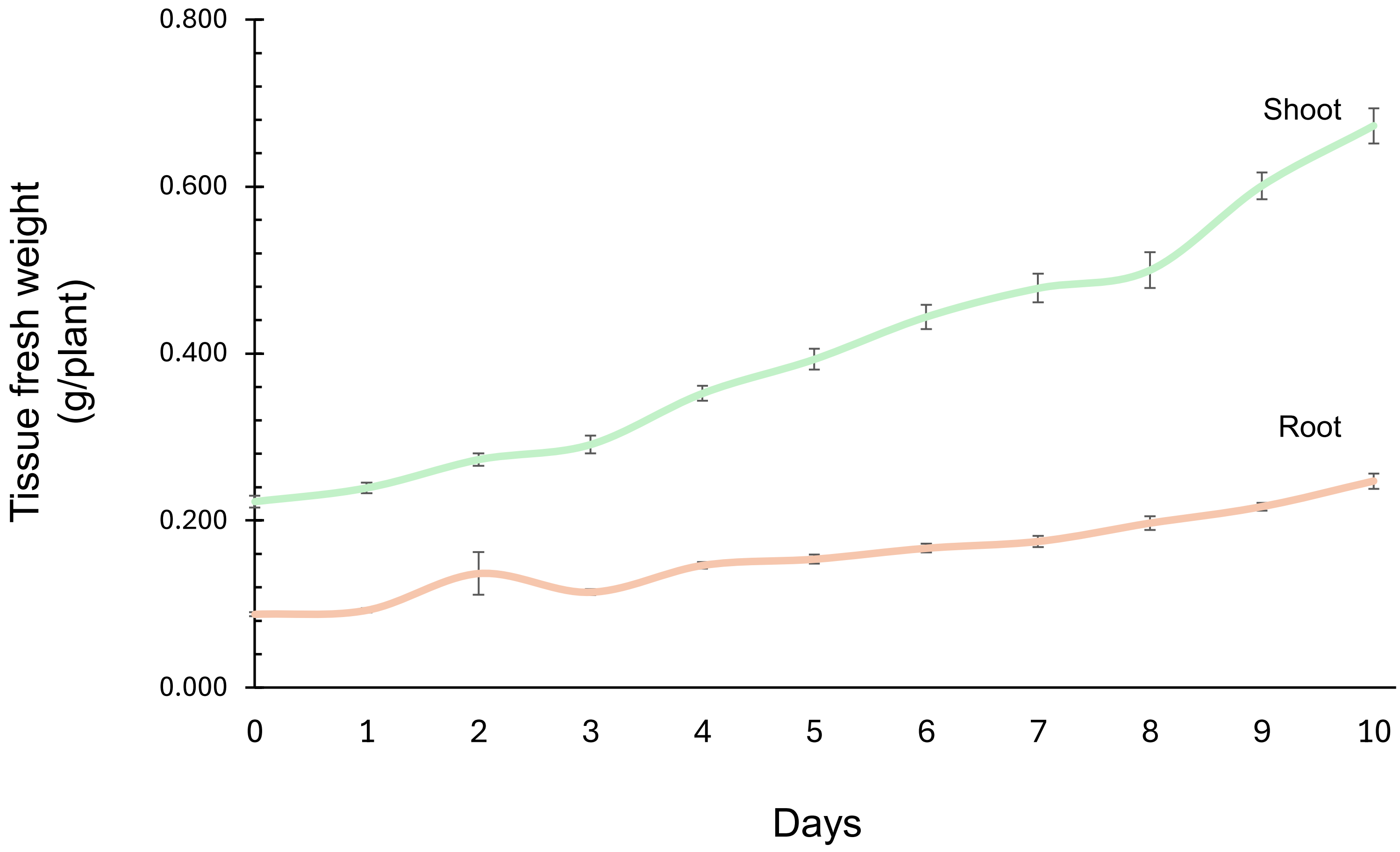

Figure S7: Growth kinetics of barley seedlings grown in hydroponics. Barley seedlings (*Hordeum vulgare* L. cv. SU MIDNIGHT; winter barley) were grown in hydroponics. Seeds were germinated on vermiculite and seedlings grown on normal growth media before transfer to treatment conditions at 8 d age. Change in shoot (**A**) and root (**B**) fresh weight was observed every day for 10 days parallel to the treatment age and time under control conditions. Data presented are means±SE, n = 3 (10 plants per experiement).

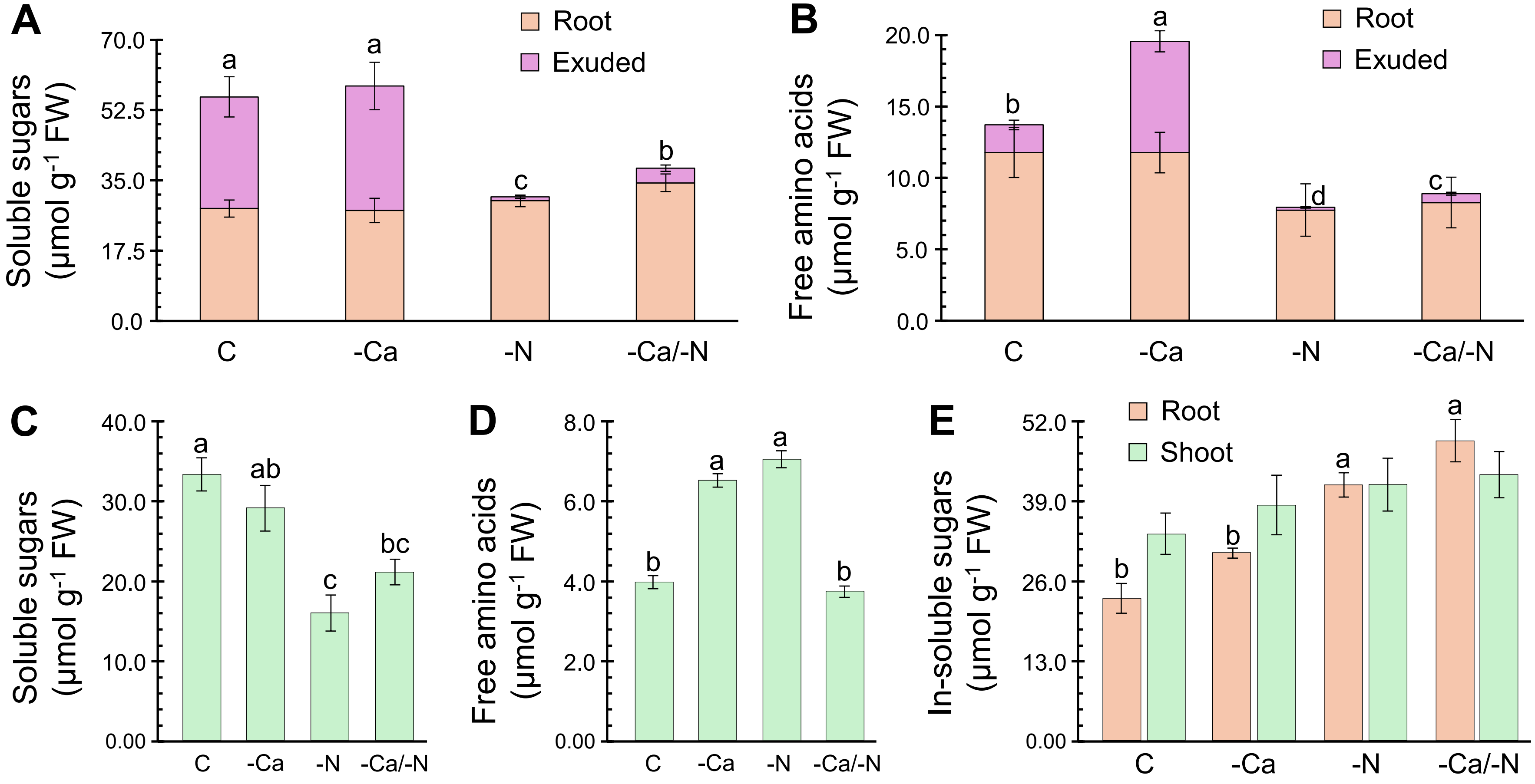

Figure S8: Sugar and amino acid contents of tissue and exudates in/from barley seedlings grown on -N, -Ca and –Ca/-N. Barley seedlings (*Hordeum vulgare* L. cv. SU MIDNIGHT; winter barley) were grown in hydroponics -N, -Ca and –Ca/-N. Seedlings were germinated on vermiculite and grown on normal growth media before transfer to treatment conditions at 8 d age. After 10 d of growth on the mentioned nutritional variability, root/shoot sugar and free amino acid analysis was carried out in fresh plant samples along with analysis of plant exudation over 6 h on the 10 d after treatment start. A comparison of root soluble sugar to that of exuded sugar (**A**), or root free amino acids to exuded amino acids (**B**), as well as shoot soluble sugars (**C**), free amino acids (**D**), and root, shoot insoluble sugar (**E**) is provided. Values are means±SE (n = 6-9 for **A-B,** 12 for **C**, 9 for **D**, 9-12 for **E,** one way ANOVA, Tukey's post hoc test, $p<0.05$ for significant differences). Different letters denote statistical differences among the four treatments for respective parameter values.

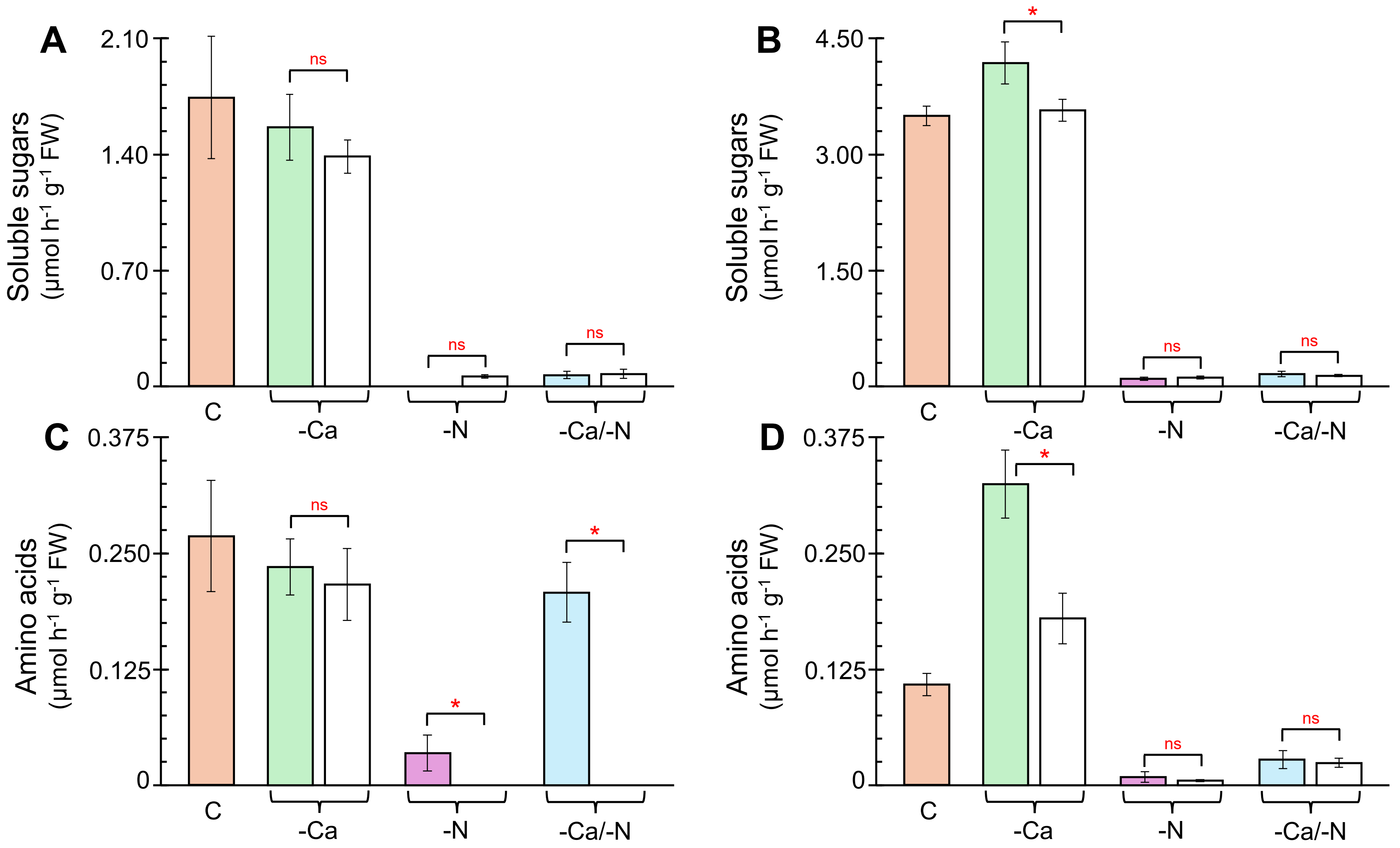

Figure S9: Effects of ionic adjustments (for missing Na/Cl ions) for –N and/or –Ca-treatments on root exudation over the treatment period. Barley seedlings (*Hordeum vulgare* L. cv. SU MIDNIGHT; winter barley) were grown in hydroponics with -N, -Ca and -Ca/-N for 10 d. NaCl is added to correct ionic imbalance: 3.5 mM NaCl for –N (-3.5 $NaNo_3$), 2 mM NaCl for –Ca (-1 mM $CaCl_2$), and 5.5 mM NaCl for -Ca/-N (all bars in white color, while colored bars are without extra NaCl). Soluble sugars (**A, B**) and amino acids (**C, D**) were quantified in root exudates after 6 h (**A**, **C**), and 10-day (**B**, **D**) of treatment. Values presented are means±SE (n = 3, 3 independent experiments, student *t*-test for comparison of ionic adjustment under individual treatments, p<0.05 for significant differences as marked by asterisks, ns = not significant). Reference samples grown on full strength media are labelled as “C”.

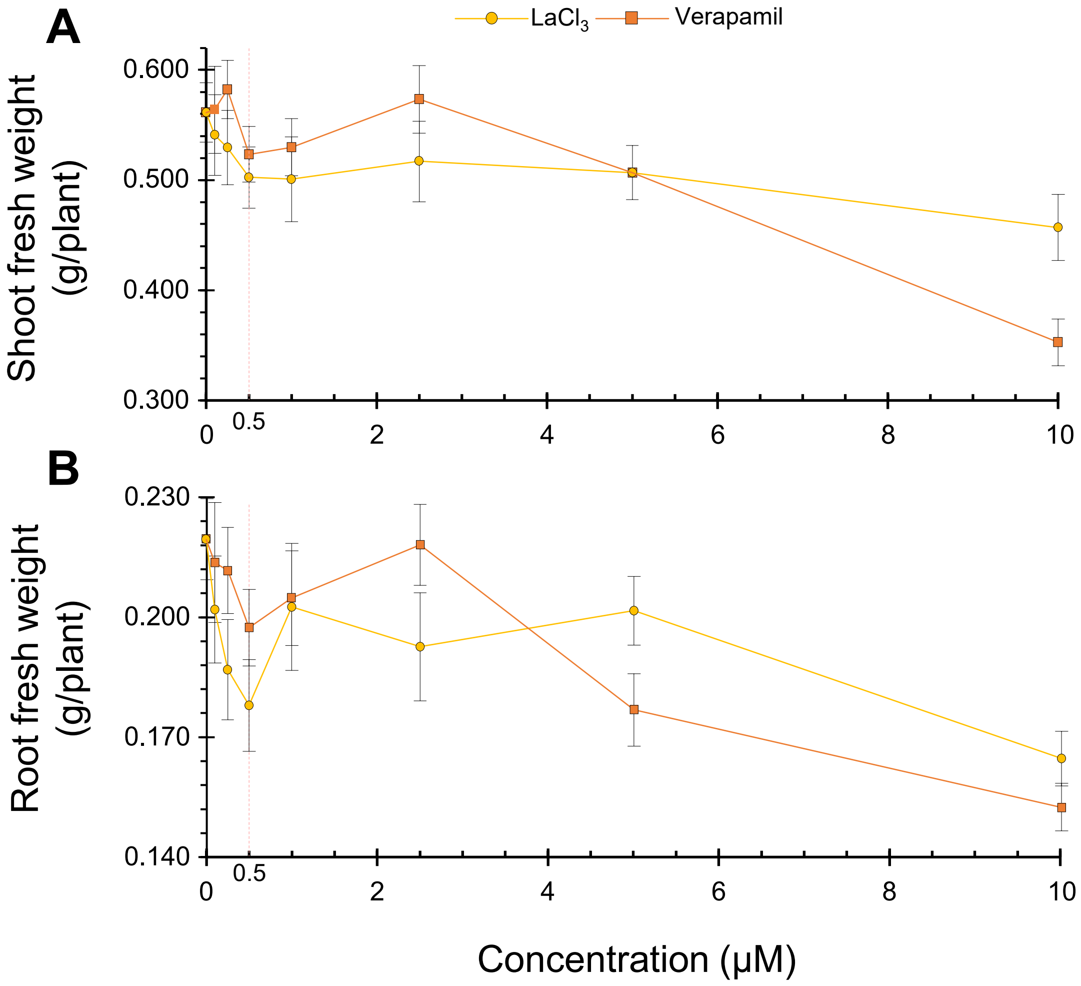

Figure S10: Plant growth responses of barley to concentration series of different Ca^{2+} channel inhibitors. Barley seedlings (*Hordeum vulgare* L. cv. SU MIDNIGHT; winter barley) were grown in hydroponics with a La^{3+} and verapamil concentration series (0-10 μM) for 10 d. Seedlings were germinated on vermiculite and grown on normal growth media before transfer to treatment conditions at 8 d age. Fresh weight of shoot (**A**) and root (**B**) in given. Values presented are means±SE (n = 8-24 plants).

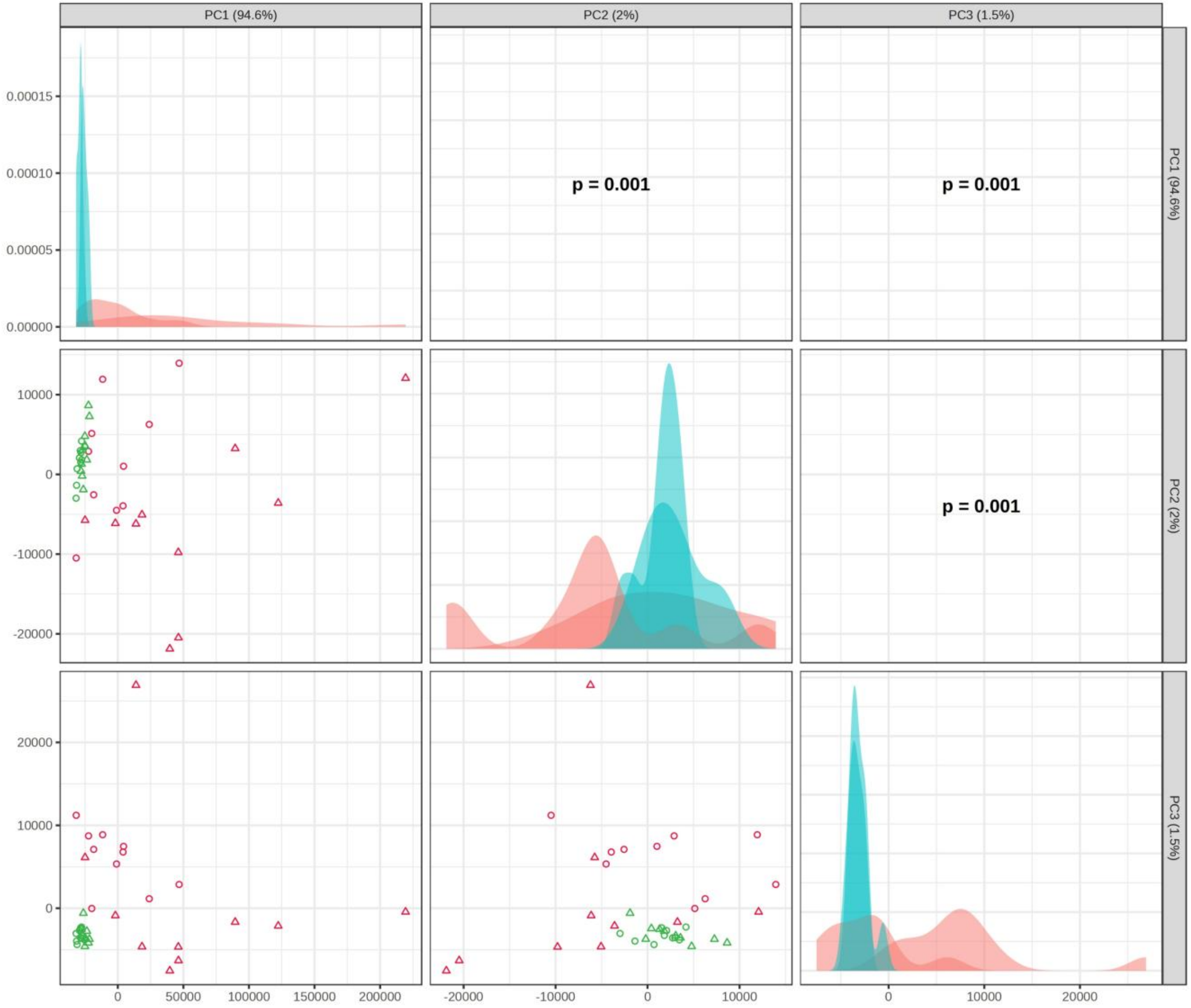

Figure S11: Principal component analysis (PCA) of metabolites quantified in barley root exudates under -N, Ca- and –Ca/-N. Barley seedlings (*Hordeum vulgare* L. cv. SU MIDNIGHT; winter barley) were grown in hydroponics with –N, -Ca and –Ca/-N. Seeds were germinated on vermiculite and seedlings grown on normal growth media before transfer to treatment conditions at 8 d age. Metabolites quantified in root exudates are subjected to PCA analysis using online tool *MetaboAnalyst* (v6.0; MetaboAnalyst). The PCA analysis in the figure panels shows distribution of individual treatment samples across top two principal components that represented maximum variability. The value for separation of samples for 4 treatment groups is higher across x- than y-axis.

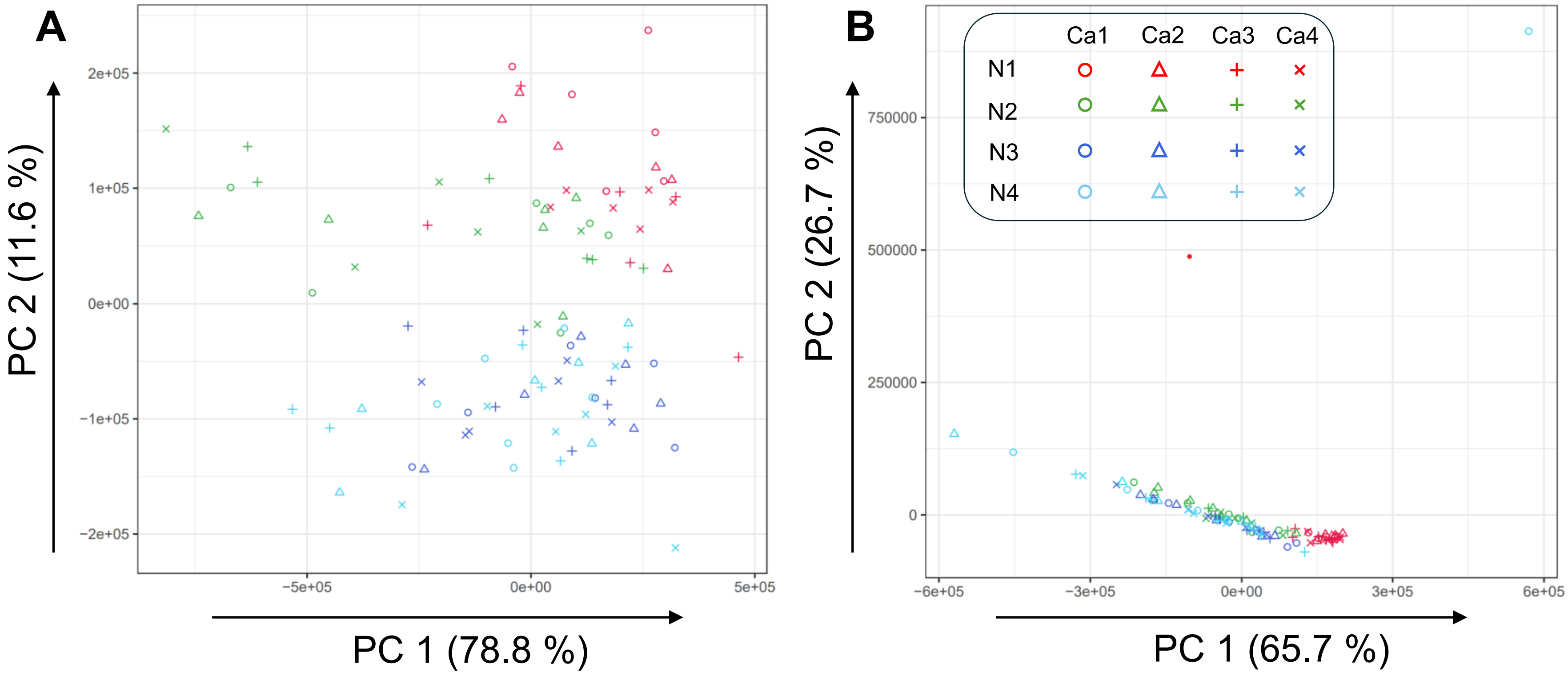

Figure S12: Principal component analysis (PCA) of metabolites quantified in barley shoot and root. Barley seedlings (*Hordeum vulgare* L. cv. SU MIDNIGHT; winter barley) were grown in hydroponics with an array of N (N1: 3.5, N2: 0.58, N3: 0.016, and N4: 0 mM), and Ca (Ca1: 1, Ca2: 0.0625, Ca3: 0.005, and Ca4: 0 mM) concentrations. Seeds were germinated on vermiculite and seedlings grown on normal growth media before transfer to treatment conditions at 8 d age. Metabolites quantified in shoot (**A**) and root (**B**) are subjected to PCA analysis using online tool *MetaboAnalyst* (v5.0; MetaboAnalyst). The PCA analysis in the figure panels shows distribution of individual treatment samples across top two principal components that represented maximum variability. The value for separation of samples for 16 treatment groups is higher across x- than y-axis.
